# Rare variation in non-coding regions with evolutionary signatures contributes to autism spectrum disorder risk

**DOI:** 10.1016/j.xgen.2024.100609

**Published:** 2024-07-16

**Authors:** Taehwan Shin, Janet H.T. Song, Michael Kosicki, Connor Kenny, Samantha G. Beck, Lily Kelley, Irene Antony, Xuyu Qian, Julieta Bonacina, Frances Papandile, Dilenny Gonzalez, Julia Scotellaro, Evan M. Bushinsky, Rebecca E. Andersen, Eduardo Maury, Len A. Pennacchio, Ryan N. Doan, Christopher A. Walsh

**Affiliations:** 1 Division of Genetics and Genomics, Boston Children’s Hospital, Boston, MA 02115, USA; 2 Department of Pediatrics, Harvard Medical School, Boston, MA 02115, USA; 3 Allen Discovery Center for Human Brain Evolution, Boston, MA 02115, USA; 4 Department of Neurology, Harvard Medical School, Boston, MA 02115, USA; 5 Howard Hughes Medical Institute, Boston Children’s Hospital, Boston, MA 02115, USA; 6 Environmental Genomics & System Biology Division, Lawrence Berkeley National Laboratory, Berkeley, CA 94720, USA

**Keywords:** autism spectrum disorder, noncoding regions, human accelerated regions, VISTA enhancers, conserved neural enhancers, caMPRA, consanguineous families, IL1RAPL1, OTX1, SIM1

## Abstract

Little is known about the role of non-coding regions in the etiology of autism spectrum disorder (ASD). We examined three classes of non-coding regions: human accelerated regions (HARs), which show signatures of positive selection in humans; experimentally validated neural VISTA enhancers (VEs); and conserved regions predicted to act as neural enhancers (CNEs). Targeted and whole-genome analysis of >16,600 samples and >4,900 ASD probands revealed that likely recessive, rare, inherited variants in HARs, VEs, and CNEs substantially contribute to ASD risk in probands whose parents share ancestry, which enriches for recessive contributions, but modestly contribute, if at all, in simplex family structures. We identified multiple patient variants in HARs near *IL1RAPL1* and in VEs near *OTX1* and *SIM1* and showed that they change enhancer activity. Our results implicate both human-evolved and evolutionarily conserved non-coding regions in ASD risk and suggest potential mechanisms of how regulatory changes can modulate social behavior.

## Highlights

• Rare variants in human-evolved and conserved non-coding regions contribute to ASD
• Contribution varies with family structure (strongest in a consanguineous cohort)
• Human accelerated regions contribute more robustly to ASD than conserved regions
• Patient variants affect enhancer activity *in vitro* and *in vivo*

## Graphical abstract

**Figure:**
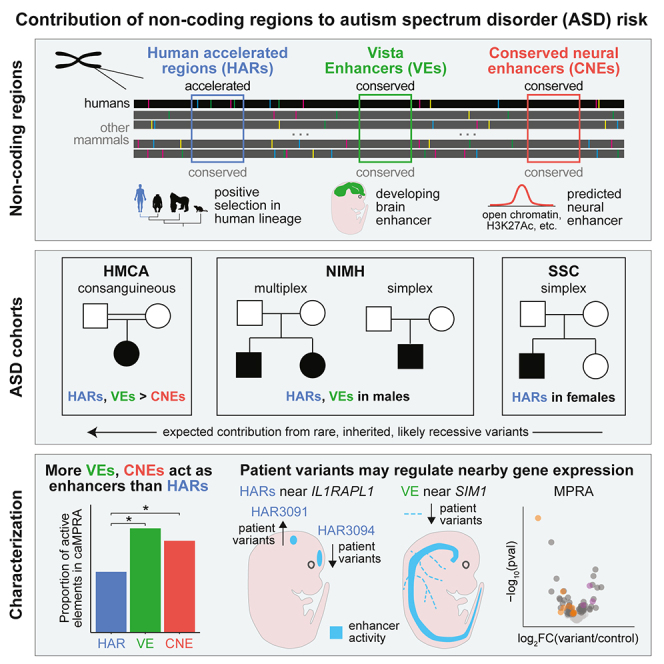

## Teaser

Shin and Song et al. demonstrate that rare, inherited, likely recessive variants in conserved brain enhancers and genomic regions under selection in humans contribute to risk for autism spectrum disorder (ASD). Patient variants regulate both ASD-associated genes (e.g., *IL1RAPL1*, *OTX1*) and genes that were not previously linked to ASD (e.g., *SIM1*).

## Introduction

Autism spectrum disorder (ASD) is a highly heritable, phenotypically complex condition that affects 2%–3% of children^1^ and shares comorbidity with many conditions, including intellectual disability, attention-deficit/hyperactivity disorder, and obesity.^2^ Over the past decade, immense progress has been made in understanding the genetic underpinnings of ASD. This has been largely driven by investigating *de novo* coding variants^3^^,^^4^^,^^5^ and, more recently, rare, recessive, inherited coding variants^5^^,^^6^^,^^7^ of moderate to large effect size. Together, these efforts have identified more than 1,000 candidate genes,^8^ with many identified ASD genes converging on similar gene programs, including synapse formation and maintenance, chromatin remodeling, and cytoskeletal pathways.^3^^,^^7^^,^^9^

Despite advances in understanding the role of coding variation in ASD, little is known about the role of non-coding variation. One major obstacle is that 98.5% of the genome is non-coding, and a systematic analysis of the entire non-coding genome requires a commensurately larger sample size to reach statistical significance. To address this issue, a number of studies have reduced the non-coding sequence search space to focus on non-coding regions that are likely to be functional and then queried whether specific classes of non-coding regions are enriched for patient variants. Evolutionary conservation has emerged as a strong marker of likely functional regions; many conserved non-coding regions are known to function as developmental enhancers, and disease-associated variants in these regions have been shown to disrupt gene regulation during development.^10^ Indeed, recent studies found that *de novo* variants in conserved promoters are enriched in patients with ASD^11^ and that *de novo* variants in conserved fetal brain enhancers are enriched in patients with severe neurodevelopmental disorders.^12^ Consanguineous families, which are enriched for recessive contributions because of shared ancestry, have also proved powerful for identifying the contribution to ASD of non-coding regions, including inherited, homozygous deletions, which have not been detectable in non-consanguineous families.^13^^,^^14^

Concurrently, multiple studies suggest that non-coding regions that show evolutionary signatures of selection in humans may be preferentially vulnerable in human diseases.^15^^,^^16^^,^^17^^,^^18^^,^^19^ For instance, human accelerated regions (HARs) are regions that are highly conserved across species, but show signs of positive selection in the human evolutionary lineage.^20^^,^^21^^,^^22^^,^^23^^,^^24^^,^^25^ HARs have been found to be enriched near genes associated with brain development,^15^^,^^26^^,^^27^^,^^28^ and rare, recessive variants in HARs are enriched in patients with ASD in consanguineous families.^17^

## Results

### HARs, VEs, and CNEs may act as regulatory elements in the brain

**Figure 1:**
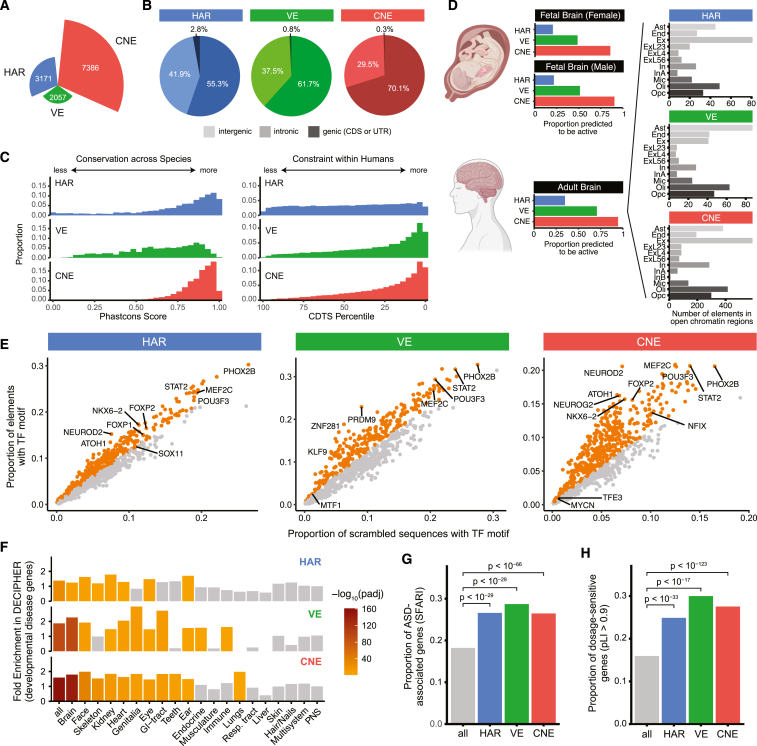
Genomic and epigenomic features of HARs, VEs, and CNEs (A) Numbers of HARs, VEs, and CNEs. (B) Proportions of HARs, VEs, and CNEs in intergenic (light coloring), intronic (moderate coloring), and genic (dark coloring) regions. (C) Conservation across species (left) and constraint within humans (right) are represented by phastCons score^31^ and CDTS percentile,^32^ respectively. (D) Proportions of HARs, VEs, and CNEs predicted to be active by ChromHMM based on epigenomic data from a fetal male brain, a fetal female brain, and an adult brain^30^ (left). Numbers of HARs, VEs, and CNEs that overlap open chromatin regions from single-cell transposome hypersensitive site sequencing (scTHS-seq) across cell types in the adult brain^33^ (right). Ast, astrocytes; End, endothelial cells; Ex, excitatory neurons; ExL23, layers 2–3 excitatory neurons; ExL4, layer 4 excitatory neurons; ExL56, layers 5–6 excitatory neurons; In, inhibitory neurons; InA, inhibitory neurons subtype A; InB, inhibitory neurons subtype B; Mic, microglia; Oli, oligodendrocytes; Opc, oligodendrocyte precursor cells. (E) Enrichment of TF-binding-site motifs in HARs, VEs, and CNEs (STAR Methods). Orange dots indicate significantly enriched elements, as assessed with the hypergeometric test at 5% false discovery rate (FDR). (F) Enrichment of HARs, VEs, and CNEs near genes associated with developmental diseases in different body systems from the DECIPHER Consortium^34^ by the binomial test at 5% FDR. (G) HARs, VEs, and CNEs are enriched for ASD-associated genes annotated in the SFARI database^8^ by the binomial test at 5% FDR. (H) Genes near HARs, VEs, or CNEs are enriched for genes with pLI >0.9 (loss-of-function intolerant)^35^ by the hypergeometric test at 5% FDR. Full details of statistical analyses are in STAR Methods.

Based on these prior studies suggesting that regions that are highly conserved or under selection in humans may be selectively vulnerable in neurodevelopmental diseases,^11^^,^^12^^,^^17^ we investigated three classes of non-coding regions for their contributions to ASD risk (Figure 1; Table S1): (1) HARs, which are regions conserved through other mammals that are likely under positive selection in humans^25^ and which were previously shown to have elevated rates of rare, recessive variants in a consanguineous ASD cohort^17^; (2) neural VISTA enhancers (VEs), which are conserved elements that have been experimentally tested to drive reporter activity in the brain in embryonic day (E) 11.5 transient transgenic reporter mice^29^; and (3) conserved neural enhancers (CNEs). We defined CNEs as elements that are highly conserved across species, are highly constrained within humans, and are predicted to be enhancers in fetal brain, neurospheres, or adult brain by ChromHMM from the Roadmap Epigenomics Project^30^ (STAR Methods). A small fraction of HARs, VEs, and CNEs overlap annotated exons, although many of these overlap both an exon and its adjacent intron (Figure 1B).

Comparison of genomic and epigenomic features of HARs, VEs, and CNEs reveals similarities and differences in conservation, mutational constraint, and predicted functional activity (Figures 1, S1, and S2). Most HARs and CNEs are highly conserved across species, whereas VEs exhibit variability in their level of conservation (Figure 1C), likely because VEs often contain conserved segments flanked by stretches of less conserved sequences.^29^ In contrast, most VEs and CNEs are highly constrained within humans and predicted to be active in fetal or adult human brain by ChromHMM,^30^ whereas HARs, which could have either gained or lost functional activity in humans, exhibit variability in their levels of mutational constraint and are less likely to be predicted to be active in fetal (∼20%) or adult (∼35%) brain (Figures 1C and 1D). Substantial proportions of HARs, VEs, and CNEs are also predicted to be active in other tissues by ChromHMM (Figure S1) and have differing cell-type specificity in the adult brain^33^ (Figure 1D). Additionally, HARs, VEs, and CNEs are enriched for transcription factor (TF) binding sites of known neurodevelopmental TFs (Figure 1E), including FOXP2 in HARs and CNEs^36^ and ZNF281 in VEs,^37^ although only CNEs are enriched in aggregate for the motifs of TFs involved in neural functions (Table S1). HARs (*p* = 0.005), VEs (*p* = 0.022), and CNEs (*p* < 10^−24^) are all enriched near genes specifically expressed in the brain in RNA sequencing data from the GTEx Consortium^38^ (STAR Methods).

### HARs, VEs, and CNEs are enriched near ASD-associated and dosage-sensitive genes

We might expect that if HARs, VEs, and CNEs modulate ASD risk, they would directly regulate the expression of genes previously implicated in ASD or other neurodevelopmental disorders. We find that HARs, VEs, and CNEs are enriched near genes implicated in severe developmental disorders that affect the brain, as annotated by the DECIPHER Consortium^34^ (Figure 1F). We also observe a strong enrichment of HARs, VEs, and CNEs near ASD-associated genes, as annotated by SFARI^8^ (adjusted *p* < 10^−29^; Figure 1G).

Given the restricted effect of a single regulatory element on gene expression,^39^^,^^40^ non-coding regions that contribute to ASD risk might preferentially regulate genes that are dosage-sensitive, i.e., genes where a small change in expression can lead to a phenotypic outcome. As a measure of dosage sensitivity, we examined the probability of loss-of-function intolerance (pLI).^35^^,^^41^ ASD-associated genes, which have been primarily identified from *de novo* heterozygous coding variants,^4^^,^^5^ are strongly enriched for dosage-sensitive genes, as expected (adjusted *p* < 10^−139^; Figure S3). Strikingly, HARs, VEs, and CNEs are all also significantly enriched near dosage-sensitive genes (adjusted *p* < 10^−17^; Figures 1H and S3).

### VEs and CNEs are more likely than HARs to act as enhancers in neural cells

**Figure 2:**
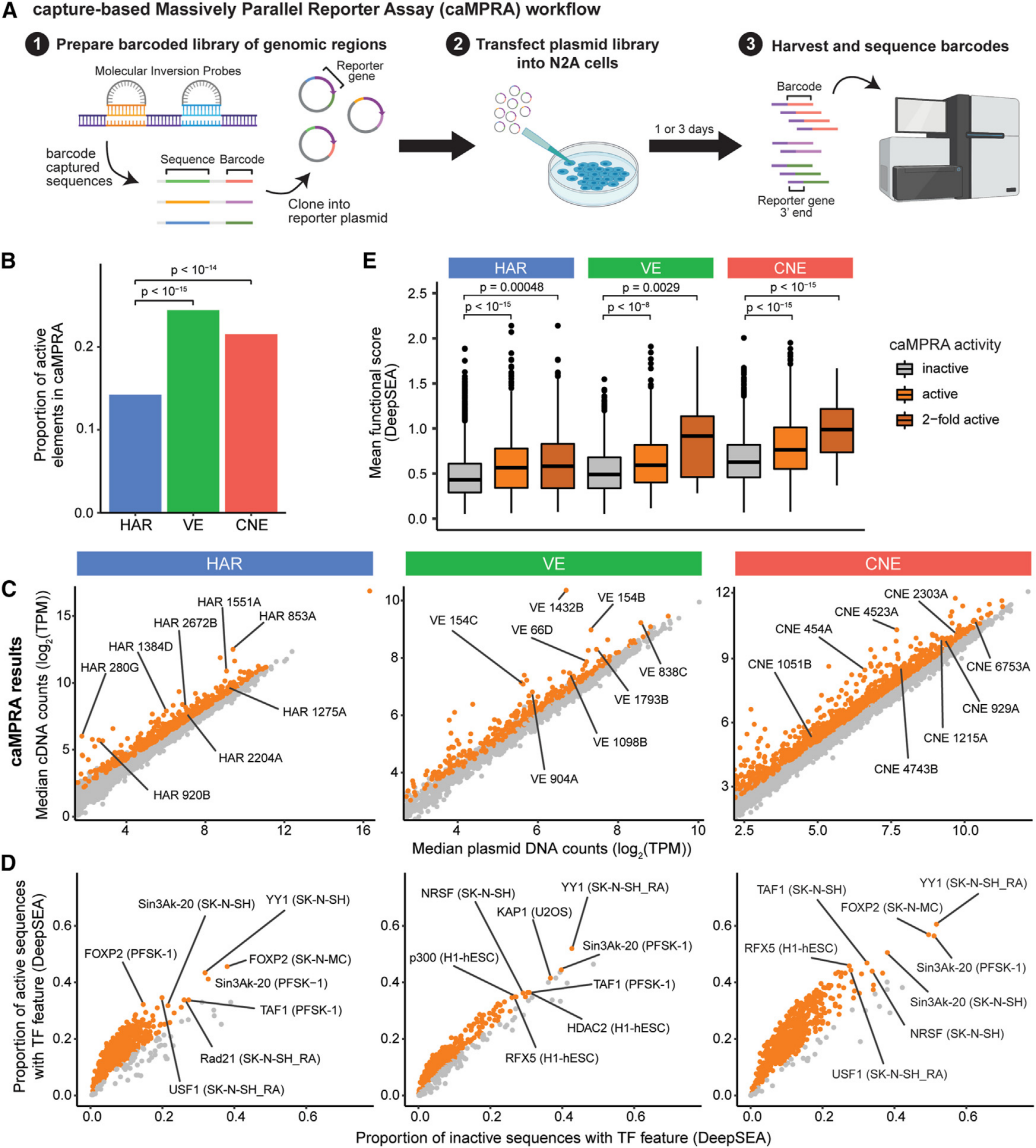
HARs, VEs, and CNEs display enhancer activity in a capture-based massively parallel reporter assay (caMPRA) (A) Schematic of caMPRA (STAR Methods). (B) Proportions of HARs, VEs, and CNEs that have enhancer activity in at least one captured sequence. Statistical significance was assessed with the chi-squared test at 5% FDR. (C) Normalized cDNA versus plasmid counts for sequences captured from HARs, VEs, and CNEs. (D) TF features were predicted by DeepSEA^45^ for each captured sequence. Representative TF features are marked in the following format: TF (cell type). (E) Sequences captured from HARs, VEs, and CNEs were classified as inactive, active, or 2-fold active and compared to their mean functional score from DeepSEA (average of −log_10_(e value) for every feature).^45^ Significant sequences are in orange and were determined by the Wilcoxon test at 5% FDR. Full details of statistical analyses are in the STAR Methods.

To directly test whether HARs, VEs, and CNEs can act as enhancers, we used a capture-based massively parallel reporter assay (caMPRA)^25^ (Figure 2A; Table S2). Unlike oligonucleotide synthesis-based MPRA methods that can only test ∼200-bp sequences cost effectively, caMPRA can test thousands of ∼500-bp sequences in parallel. This is critical because 60.6% of HARs, 34.7% of the conserved cores of VEs, and 39.3% of CNEs are >200 bp in length; conversely, 91.2% of HARs, 74.6% of conserved cores of VEs, and 92.9% of CNEs are <500 bp in length (STAR Methods; Figure S4). Using this method, we tested HARs, VEs, and CNEs for regulatory activity in Neuro2A (N2A) cells, a neuroblastoma cell line that has been previously used to assess the neural function of non-coding regions^17^^,^^42^^,^^43^^,^^44^ (STAR Methods). Enhancer activity was highly correlated across replicates (Figures S5 and S6A).

Significantly more VEs (24.4%) and CNEs (21.5%) had enhancer activity compared to HARs (14.2%) in N2A cells (*p* < 10^−14^; Figures 2B, S6B, and S6C). The proportion of HARs with enhancer activity increased slightly (15.8%) when examining only HARs predicted to be active by ChromHMM, but not significantly so. This finding is consistent with the definition of VEs and CNEs as experimentally validated or predicted conserved enhancers, respectively, whereas HARs are defined solely from genomic sequence changes in the human lineage. Active elements (Figures 2C and S6D) are enriched for the motifs of neurodevelopmental TFs, including FOXP2 and TAF1 (Figures 2D and S6E), when assessed with DeepSEA, a deep learning model trained to predict thousands of features, including TF binding.^46^ Although N2A cells were not among the cell lines used for training and prediction in DeepSEA, the TF enrichment predictions from DeepSEA for active elements were specific for cell lines similar to N2A cells, including the neuroblastoma cell lines SK-H-SH and SK-N-MC and the neuroectodermal cell line PFSK-1, demonstrating the specificity of our assay for the TF milieu present in these cells. Further, we observed strong concordance between caMPRA-based activity and the predicted functional score from DeepSEA for HARs, VEs, and CNEs, particularly for sequences that exhibit a 2-fold increase in enhancer activity by caMPRA (Figures 2E and S6F).

### High-throughput mutagenesis of HARs causes gains as well as losses of enhancer activity

We next sought to examine whether variants in these non-coding regions can affect regulatory activity. We focused on HARs and modified the caMPRA protocol to sparsely incorporate random variants into captured sequences using an error-prone PCR. Overall, we assessed 1,281 variants in 485 HARs across five replicate experiments (STAR Methods; Figures S7, S8, S9A, and S9B; Table S3). Whereas most tested variants (81.5%) did not significantly alter regulatory activity (Figures S7B and S7C) in general agreement with studies of other regulatory elements,^47^^,^^48^ we identified many variants that increased (10.8%) or decreased (7.6%) activity. These findings hold when examining only sequences that contain a single introduced random variant (Figures S9C and S9D), suggesting that single base-pair changes in HARs can have profound effects on both gains and losses of enhancer activity.

### Rare, recessive variants in HARs, VEs, and CNEs are enriched in individuals with ASD in a consanguineous cohort

To examine whether HARs, VEs, and CNEs contribute to ASD risk, we examined whether there is an excess of rare, recessive variation in HARs, VEs, and CNEs in patients with ASD. Given the redundancy of regulatory networks even for highly conserved non-coding regions,^39^^,^^40^ we reasoned that the bulk of our candidate regulatory sequences may act in a recessive manner, rather than via the *de novo* mode of contribution of highly constrained dominant genes. We first revisited a consanguineous cohort, the Homozygosity Mapping Collaborative for Autism (HMCA),^13^ where we had previously observed an excess of rare, recessive variants in HARs in ASD cases compared to controls using targeted sequencing.^17^ This enrichment was seen only when examining rare variants that were predicted to be damaging by conservation-based variant effect predictors.^17^

**Figure 3:**
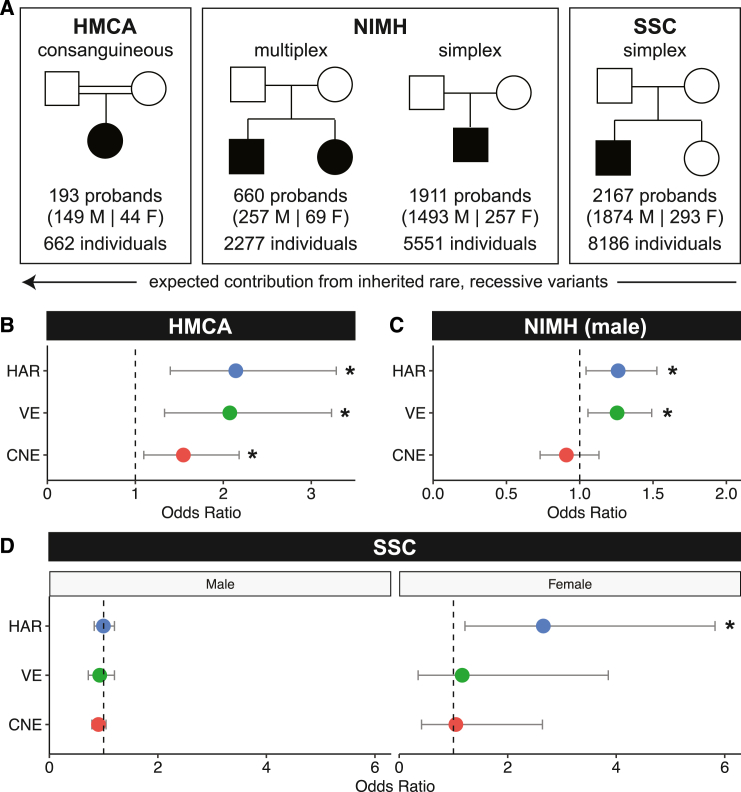
Contribution of rare, recessive variants in HARs, VEs, and CNEs to ASD varies across cohorts based on family structure (A) ASD cohorts. (B) In the HMCA cohort, cases are enriched for rare, recessive variants in HARs (adjusted *p* = 0.0014), VEs (adjusted *p* = 0.0038), and CNEs (adjusted *p* = 0.0412) at allele frequency (AF) < 0.005. (C) In the NIMH cohort, male cases are enriched for rare, recessive variants in HARs (adjusted *p* = 0.0495) and VEs (adjusted *p* = 0.0297) at AF < 0.001. (D) In the SSC cohort, female cases are enriched for rare, recessive variants in HARs (adjusted *p* = 0.0438) at AF < 0.005. All analyses were done on conserved bases. Odds ratios and 95% confidence intervals were calculated as previously described,^49^ and *p* values comparing odds ratios were calculated using *z* values assuming deviation from a normal distribution. Full details of statistical analyses are in STAR Methods.

When we now examine an expanded set of 3,171 HARs using whole-genome sequencing (WGS) on a larger number of families from HMCA (a total of 662 individuals, including 193 probands) (Figure 3A), we continue to identify a strong enrichment of rare, recessive variants in HARs in cases compared to matched controls (odds ratio [OR] = 2.142, adjusted *p* = 0.001; Figure 3B). We defined recessive variants as variants that are homozygous, compound heterozygous, or hemizygous (specifically in male individuals for the X chromosome). Because hemizygous variants on the male X chromosome are much more likely to arise compared to homozygous variants on the female X chromosomes, we examined only the autosomes when calculating combined rates for males and females, but included the X chromosome when analyzing males and females separately. As in the prior study, we observed an enrichment only when examining rare variants that are predicted to be damaging by conservation-based variant effect predictors (STAR Methods; referred to hereafter as “conserved bases”), but not when examining non-conserved bases (adjusted *p* > 0.05).

VEs also had a large excess of rare, recessive variants in conserved bases when comparing cases to controls that was similar in magnitude to the excess seen in HARs (OR = 2.074, adjusted *p* = 0.004; Figure 3B), whereas CNEs had a significant, but less pronounced, excess of rare, recessive variants in cases compared to controls (OR = 1.546, adjusted *p* = 0.041; Figure 3B). The enrichment of rare, recessive variants in HARs, VEs, and CNEs is stable across a range of low allele frequencies, suggesting that the signal we observe is not dependent on specific allele frequency cutoffs (Figure S10A). Although we are underpowered to assess significance when each sex is analyzed separately, similar excesses in rare, recessive variants were observed in both males and females (Figure S10B).

The observed rates of rare, recessive variants between cases and controls (0.239 versus 0.128 for HARs, 0.216 versus 0.117 for VEs, 0.414 versus 0.313 for CNEs) yield substantial estimated contributions to ASD of 9.9%, 11.1%, and 10.0% for recessive alleles in HARs, VEs, and CNEs, respectively (STAR Methods). Together with a previous finding in this cohort of an ∼4-fold excess of rare, homozygous, inherited deletions in non-coding, but not in coding, genomic regions,^14^ our results suggest that homozygous non-coding variation in this cohort contributes significantly to ASD risk by several mechanisms and is also consistent with a relatively modest contribution of recessive exonic mutations in this cohort.^50^

### Rare, recessive variants in HARs and VEs are enriched in individuals with ASD in non-consanguineous cohorts

We then examined whether the enrichment of rare, recessive variants in HARs, VEs, and CNEs is also observed in a larger, non-consanguineous cohort from the NIMH repository. We expect effect sizes for recessive variants to be considerably smaller in non-consanguineous cohorts compared to consanguineous cohorts, where both direct consanguinity and endogamy make it more likely that the same rare variant is inherited from both parents.^51^ However, compared to the slightly fewer than 200 probands in the consanguineous cohort, the NIMH repository contains >2,000 affected probands, offering greater resolution to detect small differences in recessive variants and to identify a larger set of patient variants for functional studies (Figure 3A). We examined 660 probands from multiplex families, where inherited variants are more likely to play a role,^7^^,^^52^ and 1,911 probands from families with only one affected child (either with or without an unaffected sibling). The latter are likely to be simplex families, where recessive variants have a lower contribution to disease compared to *de novo* mechanisms.^53^^,^^54^

Targeted sequencing of HARs, VEs, and CNEs with molecular inversion probes (STAR Methods) showed a non-significant excess of rare, recessive variants in HARs and VEs at conserved bases when considering males and females jointly (HARs, OR = 1.196, adjusted *p* = 0.186; VEs, OR = 1.193, adjusted *p* = 0.069; Figure S11A), while males considered alone, which captures hemizygous variants on the X chromosome, revealed significant enrichment for rare, recessive variants in both HARs and VEs at conserved, but not at less conserved, bases (HARs, OR = 1.262, adjusted *p* = 0.050; VEs, OR = 1.255, adjusted *p* = 0.030; Figure 3C). In contrast, CNEs were not enriched for rare, recessive variants in cases versus controls for males (males, OR = 0.909, adjusted *p* = 1; Figure 3C). Because females are much less likely than males to be diagnosed with ASD,^55^ we have a much smaller number of female individuals in this cohort and were underpowered to examine females alone, given the odds ratios observed in males (Figure S11B). Notably, the odds ratios are similar when comparing males and females jointly or males separately. Consistent with the effect of family structure on the contribution of recessive variants to ASD risk, we also observe a larger effect size in multiplex families (HARs, OR = 1.645; VEs, OR = 1.378) compared with likely simplex families (HARs, OR = 1.189; VEs, OR = 1.180) in males (Figure S11C). These findings are consistent across allele frequency cutoffs (Figure S12).

The rates of rare, recessive variants between male cases and controls are 0.141 versus 0.115 for HARs and 0.213 versus 0.181 for VEs, resulting in an estimated contribution of recessive alleles in HARs and VEs to 2.6% and 3.7% of ASD cases, respectively. This contribution is similar to the 3%–5% contribution of rare, recessive coding variants to ASD cases in a similar cohort.^6^

We next examined the Simon Simplex Collection (SSC), which consists of 8,186 individuals with WGS data and is specifically limited to simplex families with a single proband and unaffected siblings^56^ (Figure 3A). In such a cohort, recessive effects are expected to be minor and potentially undetectable.^50^^,^^53^ Indeed, we did not observe an excess of rare, recessive variants in HARs, VEs, or CNEs in most comparisons. However, when examining the cohort separated by sex, we found a significant excess of rare, recessive variants in HARs in female ASD cases in conserved, but not at less conserved, bases (OR = 2.657, adjusted *p* = 0.044; rate of rare, recessive variants is 0.027 in cases and 0.010 in controls for an estimated 1.7% contribution; Figure 3D) across allele frequency cutoffs (Figure S13), with no similar enrichment in males, despite there being 1,874 male probands and only 293 female probands in the SSC cohort. An enrichment in females, but not in males, may reflect the female protective effect, a phenomenon where female probands require a higher genetic burden (potentially including variants in HARs) than male probands to develop ASD,^55^^,^^57^^,^^58^ and also parallels the larger contribution to ASD of recessive coding variants in females compared to males.^6^

### Variants enriched in ASD patients implicate new genes in ASD risk

While we were underpowered to pinpoint specific HARs, VEs, or CNEs that are statistically enriched for patient variants, individual HARs, VEs, or CNEs with a numerical excess of rare, recessive variants in cases compared to controls represent potential candidates for further study, particularly since the number of controls far exceeded the number of cases in each cohort. We focused on rare, recessive variants enriched in ASD cases compared to controls in HARs, VEs, or CNEs from the HMCA cohort and in HARs or VEs from the NIMH cohort, because there is a greater expected contribution of inherited variants from those cohorts compared to the simplex SSC cohort.^50^^,^^51^^,^^53^

HARs, VEs, and CNEs enriched for variants found in cases compared to controls (hereafter called “patient variants”) are located near both ASD-associated genes and genes that have not been previously linked to ASD. Intriguingly, proteins encoded by many of the newly identified candidate genes are known to interact with proteins encoded by ASD-associated genes (Figure S14A). Proteins encoded by genes near patient variants also trend toward having more interactions than expected (*p* = 0.09). In addition, many newly identified candidate genes, as well as many genes previously associated with ASD, are also loss-of-function intolerant (blue circles indicate genes with pLI > 0.9 in Figures S14A and S14B).

Using the Genomic Regions Enrichment of Annotations Tool (GREAT),^59^ we found that patient variants are enriched near genes involved in the transmembrane transporter complex (adjusted *p* = 0.03), specifically ion channels (adjusted *p* = 0.02). We highlight a subset of HARs, VEs, and CNEs that are enriched for patient variants in Table 1 (full list in Table S4). These include ASD-associated genes, such as the glutamate receptor *GRIA3*^60^^,^^61^ near HAR3134, the transmembrane protein *IL1RAPL1*^62^^,^^63^^,^^64^ near HAR3094, and the TF *MEF2C*^65^ near VE644. In contrast, HAR3162 (near the transmembrane protein *SLITRK2*) and VE162 (near the TF *PROX1*) are located near promising candidate genes that have not been previously associated with ASD. Mutations in *SLITRK2* result in moderate to severe intellectual disability with a range of behavioral and neuropsychiatric symptoms.^66^ In E11.5 embryonic mice, we find that HAR3162 has enhancer activity in the ventral telencephalon (Figures S14C and S14D), where *SLITRK2* is expressed,^67^^,^^68^ suggesting that HAR3162 may regulate *SLITRK2* expression. Similarly, VE162 has enhancer activity in the ventral telencephalon in E11.5 embryonic mice^29^ and has been shown to physically interact with the promoter of *PROX1*,^69^ a gene that regulates interneuron differentiation in the ventral telencephalon.^67^^,^^70^

**Table 1:** Examples of HARs, VEs, and CNEs that have more variants found in cases compared to controls

Element	Cohort	Variants found in cases and not controls	Number of cases with variants	Number of controls with variants	Potential target genes	Disease and functional associations
HAR1362	NIMH	chr2:44721116 (G>A), chr2:44721350 (G>A)	2	0	*CAMKMT*, *SIX3*∗, *PREPL*	required for development of anterior neural structures (*SIX3*)^71^
HAR1479	NIMH	chr2:145978583 (G>A), chr2:145978593 (C>A)	2	0	*ZEB2*∗, *GTDC1*, *ARHGAP15*	mutations cause Mowat-Wilson syndrome (*ZEB2*)^72^
HAR3094	NIMH	chrX:30389661 (G>A), chrX:30389670 (A>G)	2	0	*NR0B1*∗, *CXorf21*, *IL1RAPL1*∗, *MAGEB1*, *MAGEB2*, *MAGEB3*	mutations associated with ASD and ID (*IL1RAPL1*)^62^^,^^63^
HAR3134	HMCA, NIMH	chrX:121796532 (T>C), chrX:121796545 (A>G)	3	0	*GRIA3*∗	mutations associated with ASD, X-linked syndromic ID, and schizophrenia (*GRIA3*)^60^^,^^61^^,^^73^
HAR3162	NIMH	chrX:143707357 (G>C), chrX:143707386 (A>G), chrX:143707399 (G>A), chrX:143707479 (G>A)	4	1	*SLITRK2*, *SLITRK4*	mutations associated with ID, DD, and neuropsychiatric symptoms (*SLITRK2*)^66^
VE15	NIMH	chr1:10797318 (T>C), chr1:10797401 (G>C)	2	0	*CASZ1*∗	mutations associated with ASD, ID, and DD (*CASZ1*)^74^
VE162	NIMH	chr1:213598724 (T>C), chr1:213598876 (A>C)	2	0	*PROX1*∗, *RPS6KC1*, *SMYD2*	regulates interneuron differentiation (*PROX1*)^70^
VE235	NIMH	chr2:63276286 (C>A)	1	0	*OTX1∗*	mutations associated with ASD^75^
VE462	NIMH	chr3:147564829 (T>C), chr3:147564920 (T>C), chr3:147565003 (T>C)	3	0	*ZIC1*∗, *ZIC4*	involved in medial telencephalon development (*ZIC1*)^76^
VE644	NIMH	chr5:87692588 (G>T), chr5:87692852 (C>T)	2	0	*MEF2C*∗, *TMEM161B*	mutations associated with ASD (*MEF2C*)^65^; mutations associated with polymicrogyria (*TMEM161B*)^77^^,^^78^
CNE6445	HMCA	chr17:67603809 (C>T)	2	0	*KCNJ16*, *MAP2K6*∗, *KCNJ2*	member of MAP/ERK pathway, which has been linked to changes in social behavior (*MAP2K6*)^79^
CNE7200	HMCA	chrX:18442211 (T>C)	2	0	*CDKL5*∗	mutations associated with Rett syndrome and epilepsy (*CDKL5*)^80^

A full list is in Table S4. Asterisks indicate genes that are loss-of-function intolerant (pLI > 0.9).^35^ Potential target genes were determined by gene proximity and by location within the same topologically associated domain.^81^^,^^82^ For HAR3162, one variant was observed in both a case and a control in HMCA and was excluded from the table. Coordinates are in hg19. ID, intellectual disability; DD, developmental disorder.

### HARs enriched for ASD patient variants regulate the neurodevelopmental gene *IL1RAPL1*

**Figure 4:**
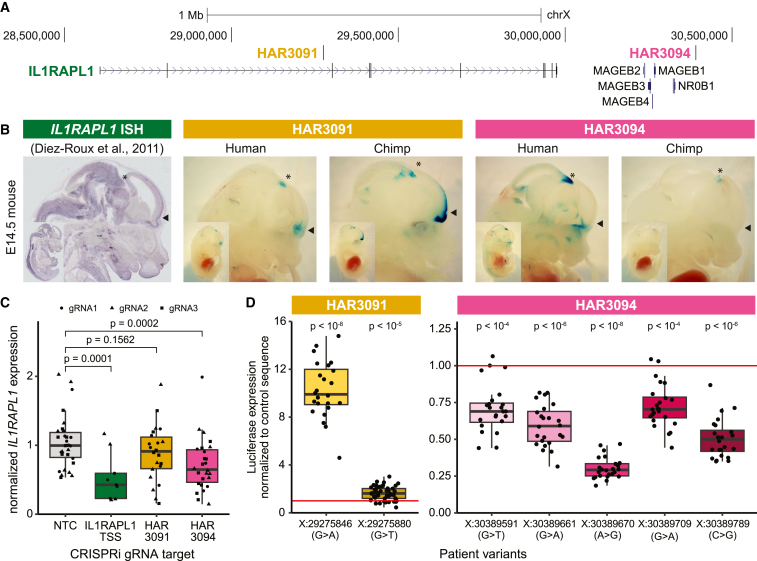
Patient variants in HAR3091 and HAR3094 likely regulate *IL1RAPL1* expression in multiple brain regions (A) Genomic interval containing *IL1RAPL1*, HAR3091, and HAR3094. (B) Constructs containing either the human or the chimpanzee version of HAR3091 and HAR3094 cloned upstream of a minimal promoter driving lacZ expression were randomly integrated into mice and analyzed at E14.5. Representative embryos are shown (all embryos are in Figure S15). Arrowheads, telencephalon and olfactory bulb; asterisks, midbrain. E14.5 embryos have an average crown-rump length of 12 mm. *In situ* hybridization of *IL1RAPL1* at E14.5 from the Eurexpress database^68^ is shown for comparison. (C) CRISPRi targeting the transcription start site (TSS) of *IL1RAPL1*, HAR3091, and HAR3094 compared to non-targeting control (NTC) gRNAs in iPSC-derived neurons. Statistical significance was determined with the Wilcoxon test and Fisher’s method at 5% FDR. (D) Patient variants in HAR3091 and HAR3094 were tested for luciferase expression in N2A cells. Statistical significance was determined with the Wilcoxon test and Fisher’s method at 5% FDR. Coordinates are in hg19. Full details of statistical analyses are in the STAR Methods.

As an initial functional investigation into whether HARs, VEs, or CNEs that are enriched for patient variants might contribute to ASD risk, we characterized HAR3091 and HAR3094 (Figure 4A). Both HARs are within the same topologically associated domain as *IL1RAPL1*,^83^ act as enhancers by caMPRA, and are enriched for patient variants. *IL1RAPL1* is a loss-of-function-intolerant gene^35^^,^^41^ important for synaptic density and dendrite formation at excitatory synapses.^64^ Exonic point mutations, deletions, and duplications of *IL1RAPL1* have been associated with ASD and intellectual disability,^34^^,^^64^^,^^84^^,^^85^^,^^86^^,^^87^^,^^88^^,^^89^^,^^90^ suggesting that *IL1RAPL1* is dosage-sensitive to both gain and loss of expression. In the NIMH cohort, HAR3091 and HAR3094 each contained two variants in cases and none in controls at conserved bases (Tables 1 and S4).

To examine HAR3091 and HAR3094 enhancer activity, we assessed mice injected with either the human or the chimpanzee version of HAR3091 or HAR3094 upstream of a minimal promoter driving the *lacZ* reporter gene at E14.5, when *in situ* data show strong *IL1RAPL1* expression^68^ (STAR Methods; Data S1; Figures 4B and S15). The chimpanzee version of HAR3091 drives lacZ expression predominantly in the telencephalon and olfactory bulb (arrowheads in Figures 4B and S15) and is more robust than the human version of HAR3091. In contrast, the human version of HAR3094 drives lacZ expression predominantly in the midbrain (asterisks in Figures 4B and S15) and is more robust than the chimpanzee version of HAR3094. This suggests that HAR3091 is primarily a telencephalon enhancer that has decreased activity in humans compared to chimpanzees, whereas HAR3094 is primarily a midbrain enhancer that has increased activity in humans compared to chimpanzees. Both HAR3091 and HAR3094 enhancer domains overlap with regions where *IL1RAPL1* is expressed at E14.5^68^ (Figure 4B).

To directly test whether HAR3091 and HAR3094 might regulate *IL1RAPL1* expression, we used CRISPR inhibition (CRISPRi), where a nuclease-inactive Cas9 variant tethered to a KRAB domain (dCas9-KRAB) heterochromatizes and silences the target region.^91^ We induced *NGN2* expression (STAR Methods) to differentiate human induced pluripotent stem cells (iPSCs) into a heterogeneous mixture of excitatory neurons that resemble neurons derived from multiple brain regions, including the regions where HAR3091 and HAR3094 have enhancer activity.^92^ Targeting the *IL1RAPL1* transcription start site (TSS) significantly decreased *IL1RAPL1* expression compared to non-targeting control (NTC) guide RNAs (gRNAs), as expected (adjusted *p* = 0.0001; Figure 4C). We also observed a significant decrease in *IL1RAPL1* expression when targeting HAR3094 (adjusted *p* = 0.0002), suggesting that HAR3094 acts as an *IL1RAPL1* enhancer. When HAR3091 was targeted, median *IL1RAPL1* expression decreased nominally by 8.8% but did not reach statistical significance. Given that human HAR3091 acts as a weak enhancer in transgenic mice, it is possible that our CRISPRi assay lacked the required sensitivity to detect a significant decrease in expression, especially given the wide variability in gRNA efficacy (Figure S16).

Next, we asked whether patient variants may affect the enhancer activity of HAR3091 and HAR3094. Based on the availability of patient DNA, we examined one of the two rare, recessive patient variants at conserved bases in HAR3091 and the two rare, recessive patient variants at conserved bases in HAR3094. In addition, we also examined additional patient variants that are rare, recessive variants but at less conserved bases. HAR3091 or HAR3094 sequences containing these variants were cloned upstream of a minimal promoter driving luciferase expression, and luciferase activity was assessed in N2A cells (STAR Methods). Strikingly, we found that patient variants for HAR3091 significantly increased luciferase activity compared to the control HAR3091 sequence and that patient variants for HAR3094 significantly decreased luciferase activity compared to the control HAR3094 sequence (Figures 4D and S17). The largest effect sizes were observed for the patient variants at conserved bases, consistent with the established link between conservation and functional activity and our finding that an excess of rare, recessive variants is observed in ASD cases compared to controls for conserved but not less conserved bases. TF motif analysis further revealed TF binding sites that may be gained or lost due to patient variants (Table S4), including the creation of a binding site for the transcriptional repressor RUNX3 by the A>G variant at chrX:30389670. These results indicate that patient variants modulate HAR3091 and HAR3094 enhancer activity and may result in changes to *IL1RAPL1* expression in specific brain regions.

### Patient variant near *OTX1*, an ASD-associated gene, modulates *in vivo* enhancer activity

**Figure 5:**
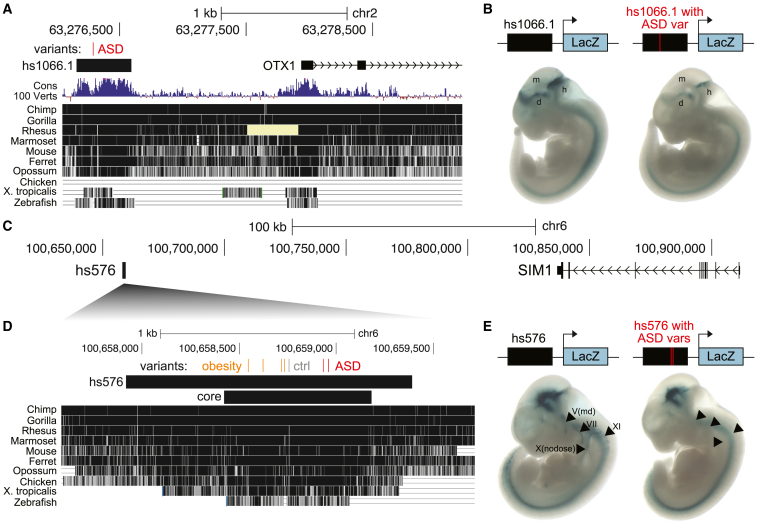
Patient variants in VISTA enhancers reduce enhancer activity in the nervous system (A–E) Genomic intervals containing hs1066.1 and *OTX1* (A) or hs576 and *SIM1* (C and D). Coordinates are in hg19. The locations of ASD patient variants are in red, control variants are in gray, and obesity-associated variants^94^ are in orange. (A) The pale yellow bar in the alignment to *Rhesus* indicates missing sequence (Ns) in that region. (D) The core region of hs576 recapitulates most of the enhancer activity of the entire element.^94^ Constructs containing hs1066.1 (B) or hs576 (E) without or with ASD patient variant(s) upstream of a minimal promoter driving the lacZ gene were integrated into the safe-harbor H11 locus and analyzed for lacZ expression at E11.5 (STAR Methods). Representative embryos are shown (all embryos are in Figures S18 and S19). E11.5 embryos have an average crown-rump length of 6 mm. (B) d, diencephalon; m, midbrain; and h, hindbrain. (E) Arrowheads indicate cranial nerves where the inclusion of the two ASD patient variants reduces enhancer activity.

To examine whether ASD patient variants can modulate enhancer activity *in vivo*, we assessed VE235, commonly referred to as hs1066.1, a highly conserved VE that has been previously shown to drive robust enhancer activity in multiple brain regions, including the diencephalon, midbrain, and hindbrain, in E11.5 mice.^29^ VE235 contains one rare, recessive ASD patient variant in the NIMH cohort and is located 1 kb from *OTX1* (Table 1; Figure 5A). Mutations in *OTX1* have been previously associated with ASD,^8^^,^^75^ and *Otx1*-null mice have abnormalities in multiple brain regions, including the hippocampus, midbrain, and hindbrain,^93^ matching the enhancer domains of hs1066.1. To test whether the ASD patient variant in hs1066.1 affects enhancer activity, we first confirmed the known expression pattern of hs1066.1 in E11.5 mice by integrating a construct containing hs1066.1 upstream of a minimal promoter driving lacZ expression at the H11 safe-harbor locus (STAR Methods; Figure 5B). We then generated E11.5 transgenic mice where hs1066.1 containing the ASD patient variant was integrated at the H11 locus. Strikingly, we found decreased enhancer activity in the diencephalon, midbrain, and hindbrain in E11.5 mice containing hs1066.1 with the patient variant (Figures 5B and S18). TF motif analysis further revealed that this patient variant removes a TF binding site for transcriptional activators in the C2H2 zinc-finger class, such as EGR1 and MAZ, while creating a TF binding site for zinc-finger repressors (Table S4). These results suggest that the patient variant in hs1066.1 may decrease expression of *OTX1*, an ASD-associated gene, by altering TF binding.

### ASD patient variants near *SIM1*, a human neurobehavioral gene, modulate *in vivo* enhancer activity in cranial nerves

Both *IL1RAPL1* and *OTX1* have been previously associated with ASD, and we uncovered patient variants in nearby non-coding regions that may modulate their expression. To assess whether our analyses can also identify non-coding variants regulating genes that have not been previously associated with ASD, we examined VE854, commonly referred to as hs576,^29^ which is an enhancer of the nearby obesity-associated gene *SIM1*^94^^,^^95^^,^^96^^,^^97^ and contains two rare, recessive patient variants in the HMCA and NIMH cohorts (Figure 5C; Table S4). *SIM1* loss of function has been associated with obesity and neurobehavioral deficits; in one study that identified 13 obese individuals with rare, *de novo SIM1* mutations, 11 also presented with neurobehavioral abnormalities including impaired concentration and emotional lability.^98^ Genes downstream of *SIM1* have similarly been associated with both obesity and neurological phenotypes,^99^ suggesting that modulating this pathway may contribute to obesity, ASD, and their comorbidity.

hs576 has been previously found to drive enhancer activity in the developing brain, somites, and cranial nerves in transgenic E11.5 mice and in the forebrain and hippocampus in E14.5 mice, matching the expression pattern of *SIM1*.^29^^,^^94^ This enhancer activity is mainly derived from the most highly conserved portion of hs576 (“core” in Figure 5D).^94^ Intriguingly, obesity-associated variants^94^ and our identified ASD patient variants are both located in this core region, albeit in separate clusters at the 5′ and 3′ ends, respectively (Figure 5D). There is also one control individual in the NIMH cohort that contains two neighboring variants in hs576 (Figure 5D). To test whether the ASD patient variants affect the enhancer activity of hs576, we first confirmed its known expression pattern in E11.5 mice by integrating a construct containing hs576 upstream of a minimal promoter driving lacZ expression at the H11 safe-harbor locus (STAR Methods; Figure 5E). We then generated E11.5 transgenic mice where hs576 containing the two ASD patient variants was integrated at the H11 locus. Strikingly, we found that hs576 containing the two ASD patient variants had reduced or absent enhancer activity in multiple cranial nerves, in particular the mandibular portion of the trigeminal nerve (V), the facial nerve (VII), the nodose nerve/inferior part of vagus nerve (X), and the accessory nerve (XI), across multiple embryos (arrowheads in Figures 5E and S19). TF motif analysis indicated that one of the ASD patient variants (chr6:100658934, C>T) abolishes a binding site for the transcriptional activator MEIS1 and creates a new binding site for the transcriptional repressor BACH2, providing a potential mechanism for action. These results suggest that ASD patient variants can alter *SIM1* expression and implicate *SIM1* in ASD etiology.

### High-throughput identification of ASD patient variants that modulate enhancer activity

**Figure 6:**
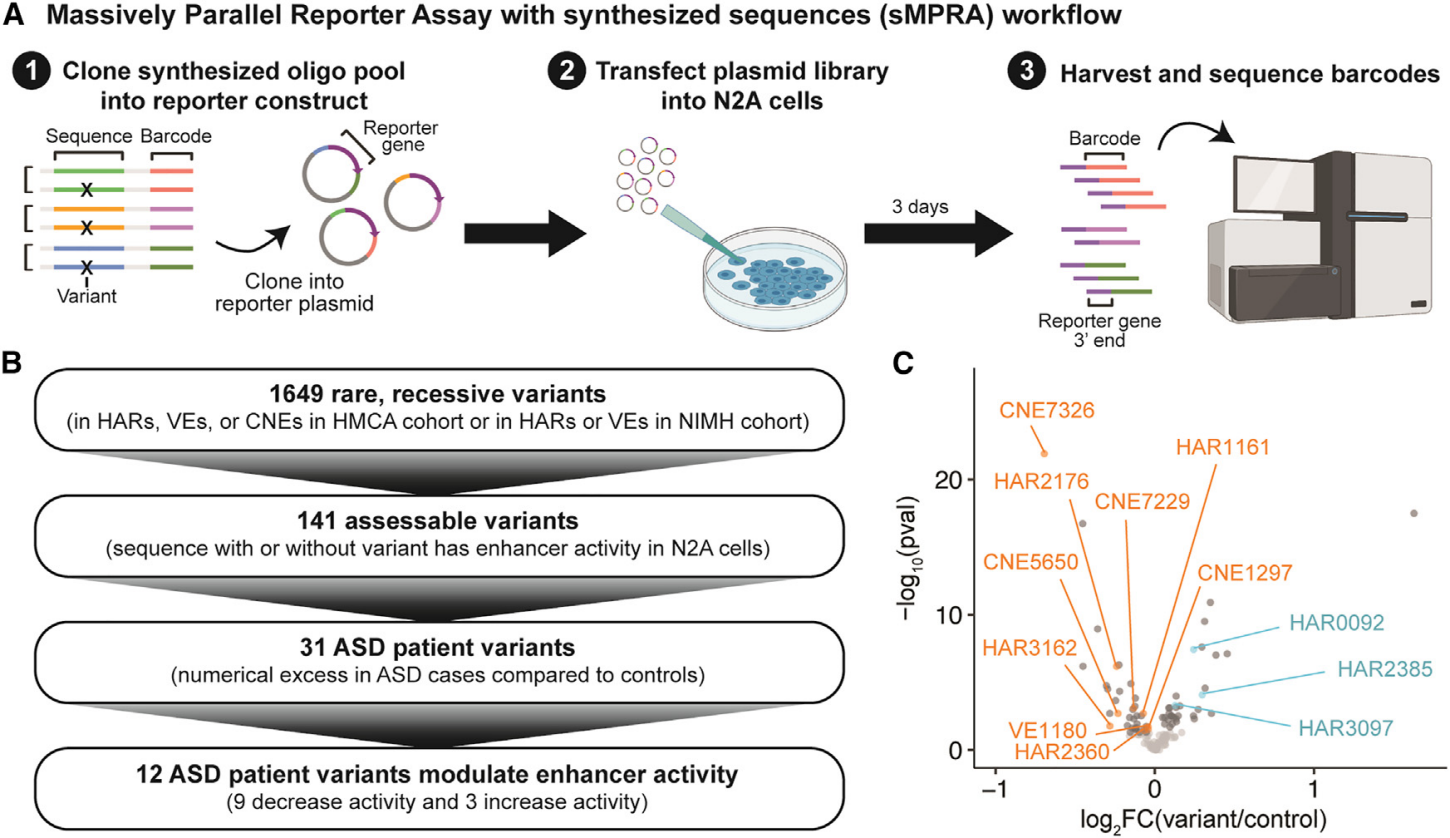
Identification of patient variants that modulate enhancer activity using MPRA with synthesized sequences (sMPRA) (A) Schematic of sMPRA. (B) Flowchart showing the number of rare, recessive variants that pass each filter. (C) Volcano plot of fold change of enhancer activity and adjusted *p* value for each variant-containing sequence compared to its matched control sequence. Significant patient variants are labeled and in color, significant control variants are in dark gray, and all other variants are in light gray. Statistical significance was assessed with the Wilcoxon test at 5% FDR. Full details of statistical analyses are in the STAR Methods.

To identify additional ASD patient variants with functional effects, we performed an MPRA in N2A cells of the 1,649 rare, recessive variants identified in HARs, VEs, or CNEs in the HMCA cohort and in HARs or VEs in the NIMH cohort from both cases and controls (STAR Methods; Table S4; Figure 6A). Because the limited availability of case and control DNA made direct capture of ∼500-bp sequences for caMPRA infeasible, we synthesized matched 238-bp sequences with or without the rare, recessive variant. We observed weak correlation between overlapping control sequences tested both by caMPRA and by MPRA with synthesized sequences (sMPRA) (Pearson r = 0.3), consistent with prior results showing that increased sequence length in MPRAs contributes substantial biological context.^100^

To restrict our analysis to variants that have enhancer activity at a shorter fragment size, we first identified 141 sequences where the sequence either with or without the variant drove significant enhancer activity (STAR Methods; Figure 6B). Of these, 31 had a numerical excess of rare, recessive variants in ASD cases compared to controls. Nine of the 31 ASD patient variants significantly decreased enhancer activity, whereas only 3 increased enhancer activity (Figure 6C). In contrast, the 110 rare, recessive variants that were not numerically enriched in ASD cases compared to controls were equally distributed between those that increased (29) and those that decreased (28) enhancer activity. The 12 ASD patient variants that modulate enhancer activity implicate both known ASD genes, such as *LNPK*^101^ near CNE1297, and novel candidate genes, such as *SLITRK2* near HAR3162 (Table S1; Figures S14C and S14D). Together with the ASD patient variants assessed in Figures 4 and 5, these variants provide additional entryways into understanding how regulatory changes contribute to ASD risk.

## Discussion

We find that HARs consistently have the highest odds ratios for rare, recessive variants in ASD compared to controls across all three cohorts, followed by VEs and then by CNEs. This may suggest that regions that are recently evolved in humans are more likely to contribute to disease risk than conserved regions. Intriguingly, two new sets of HARs that were identified after the completion of this study^102^^,^^103^ are also nominally enriched for rare, recessive variants in ASD cases compared to controls in the HMCA cohort (Figure S20). Our results extend recent findings that common variation in non-coding, human-evolved regions may contribute to risk for neurological diseases^16^^,^^18^^,^^19^ and suggest that rare variation in human-evolved regions may also preferentially contribute to human disease risk.

Further, our results suggest that HARs are more heterogeneous than VEs or CNEs in regulatory function. The proportion of HARs with enhancer activity in the caMPRA experiment was significantly lower than the proportions of VEs and CNEs with enhancer activity (Figures 1D and 2B). We also find that HARs include regions such as HAR3091 that were previously strong enhancers in chimpanzees, but where decreasing or silencing enhancer function appears to have been selected for in the human lineage, similar to findings from prior MPRAs.^25^^,^^104^^,^^105^ We also clearly observe heterogeneity in HAR function in the caMPRA mutagenesis experiment and the sMPRA patient variant experiment, where similar proportions of variants increase or decrease enhancer activity in HARs (Figures S7C and 6). In contrast, prior mutagenesis studies that examined known enhancers, including VEs, found that most functional variants decreased enhancer activity,^47^^,^^48^ and we correspondingly observed that the patient variants in VEs and CNEs that modulate enhancer activity in our sMPRA experiment and *in vivo* enhancer assays all decrease activity. In addition, when we compare the proportion of rare, recessive variants in elements that are active by caMPRA, there is a nominal enrichment for patient variants in active compared to inactive VEs (*p* = 0.052) but not so for HARs (Figure S21); this further suggests that VEs are enhancers at baseline, whereas HARs are more heterogeneous in function. These findings, together with the strong enrichment for rare, recessive variants in HARs, emphasize the importance of examining non-coding regions that perform different regulatory functions for their contributions to ASD risk.

In contrast, CNEs had the lowest odds ratios for recessive ASD risk in all cohorts, despite being more highly conserved across species, more highly constrained within humans, and more likely to be predicted to act as enhancers than HARs and VEs. This raises the possibility that variants in CNEs may act in a dominant, *de novo*, rather than recessive, inherited, fashion. Although we do not observe a case-specific enrichment of *de novo* variants in CNEs in the SSC cohort (Figure S22), it is possible that an increased sample size may reveal a *de novo* contribution.

Prior work in non-consanguineous multiplex cohorts did not detect a significant contribution of non-coding, inherited variation when examining regions predicted to be functional (using heuristics similar to those we use here to define CNEs) and suggested that sample sizes of 8,000–9,000 probands would be required for sufficient statistical power.^52^ In contrast, we find a significant enrichment for rare, recessive variants for HARs, VEs, and CNEs in a consanguineous cohort with only 193 probands and confirm this enrichment for HARs and VEs in a cohort containing multiplex families. Notably, the impact of variants in HARs, VEs, and CNEs tracks the known contribution of recessive variants as a function of family structure, with by far the largest contribution (∼10%) seen in consanguineous families, a smaller contribution from HARs and VEs (∼3%–5%) in male ASD cases in a cohort of non-consanguineous families that includes multiplex families, and a contribution from HARs (∼2%) that is only discernible in females among simplex families. This suggests (1) that HARs and VEs, which are less likely than CNEs to be predicted to be active by epigenomic data, are particularly impactful sets of non-coding regions, and current predictors of functional activity require improvement, and (2) that consanguineous families may be especially suitable for analyzing non-coding contributions to disease risk, given that both direct consanguinity and endogamy enhance potential recessive contributions.^51^

We find that proteins encoded by both ASD-associated and previously unassociated genes near patient variants are known to interact, mirroring recent studies that identify convergent effects on protein networks across multiple, distinct genetic models of ASD.^106^^,^^107^^,^^108^ Many of these previously unassociated genes are dosage-sensitive, known to play critical roles in neurodevelopment, or mutated in severe developmental disorders (Table 1; Figure S14). This suggests a model whereby coding variants in these genes lead to embryonic lethality or to multisystem developmental disorders, but non-coding variants in nearby regulatory sequences dysregulate gene expression in specific contexts to contribute to ASD risk.

Our results also identify opposing effects of patient variants in HAR3091 and HAR3094 on *IL1RAPL1* expression in different tissues. Intriguingly, *IL1RAPL1* is a dosage-sensitive gene, where both knocking out and overexpressing *Il1rapl1* in mice change excitatory synapse number.^109^^,^^110^^,^^111^^,^^112^^,^^113^ This could potentially result in an excitatory-inhibitory synaptic imbalance, one of the hallmark cellular phenotypes observed in ASD models.^114^ Future studies modeling HAR3091 and HAR3094 patient variants will be needed to understand how modulating *IL1RAPL1* expression might impact ASD risk.

Peripheral nervous system deficits have also been increasingly linked to ASD symptoms, including flat facial expressions and touch and taste sensitivity.^115^^,^^116^^,^^117^ These symptoms are likely driven, at least in part, by cranial nerves, including those affected by the patient variants in hs576, an enhancer of the obesity-associated *SIM1* gene.^98^ The vagus nerve, in particular, is also important in appetite regulation,^118^ and its dysregulation might underlie the comorbidity of obesity and ASD.^119^ Unfortunately, detailed weight information for the ASD patients containing hs576 variants was not available, so future research will be needed to determine whether these ASD patient variants affect *SIM1* expression in ways that solely contribute to neurobehavioral deficits or that may also contribute to obesity.

Collectively, these findings identify classes of non-coding regions that contribute to ASD disease risk and nominate specific non-coding elements and ASD patient variants for future study. Our work highlights the importance of examining a diverse set of non-coding regions for their contribution to disease risk, including human-evolved elements and non-coding regions with diverse regulatory functions. Further, our data also demonstrate the importance of expanding cohort enrollment to diverse populations and potentially focusing on populations with high rates of consanguinity and endogamy, since such families may be very powerful for elucidating the contribution of non-coding regions to ASD and other diseases.

### Limitations of the study

We compared only three sets of non-coding regions, and it is possible that analyzing additional regions would change the conclusions of our study regarding the relative contributions of human-evolved and conserved regions. Our MPRA and luciferase experiments were performed in mouse neuroblastoma N2A cells, which are commonly used but do not reflect a healthy, physiological cell state. We also acknowledge that differences between the mouse and the human genetic backgrounds may affect our *in vitro* and *in vivo* results.

## STAR★Methods

### Key resources table

**Table:** 

REAGENT or RESOURCE	SOURCE	IDENTIFIER
**Bacterial and virus strains**		

TOP10 Chemically Competent *E. coli*	Thermo Fisher	Cat #C404006
MegaX DH10B T1R Electrocompetent Cells	Thermo Fisher	Cat #C640003

**Biological samples**		

NA12878	Coriell Institute	NA12878
Human Genomic DNA	Promega	Cat #G1471

**Chemicals, peptides, and recombinant proteins**		

Fetal bovine serum	Atlanta Bio	Cat #S11150
Dulbecco’s Modified Eagle Medium	Thermo Fisher	Cat #MT10013CV
Penicillin-Streptomycin	Thermo Fisher	Cat #15140122
0.25% (w/v) Trypsin	Thermo Fisher	Cat #25200-114
Lipofectamine 3000	Thermo Fisher	Cat #L3000015
Lipofectamine LTX with PLUS Reagent	Thermo Fisher	Cat #15338100
AMPure XP beads	Beckman-Coulter	Cat #A63881
AsiSI restriction enzyme	NEB	Cat #R0630
PspXI restriction enzyme	NEB	Cat #R0656
SfiI restriction enzyme	NEB	Cat #R0123
MlyI restriction enzyme	NEB	Cat #R0610
Phusion DNA Polymerase	Thermo Fisher	Cat #F530L
Ampligase	VWR	Cat #A3210K
Exonuclease I	Thermo Fisher	Cat #EN0582
Exonuclease III	Thermo Fisher	Cat #EN0191
Phusion DNA Polymerase High Fidelity Master Mix	Thermo Fisher	Cat #F531L
T4 DNA Ligase	Thermo Fisher	Cat #EL0011
Trimethoprim	Sigma Aldrich	Cat #92131
Puromycin	Sigma Aldrich	Cat #P8833
TRIzol reagent	Invitrogen	Cat #15596018
Brilliant II SYBR Green Low ROX qPCR Master Mix	Agilent	Cat #600830
NEBuilder HiFi DNA Assembly Mix	NEB	Cat #E2621
Alt-R SpCas9 Nuclease V3	IDT	Cat #1081058
DirectPCR Lysis Reagent	Viagen	Cat #301-C

**Critical commercial assays**		

Neon Transfection System Kit	Thermo Fisher	Cat #MPK10025
Dynabeads mRNA DIRECT Purification Kit	Thermo Fisher	Cat #61012
QIAquick Nucleotide Removal Kit	Qiagen	Cat #28306
Qiagen Plasmid Maxi Kit	Qiagen	Cat #12162
GeneMorph II Random Mutagenesis Kit	Agilent	Cat #200550
Superscript VILO Master Mix with EZ DNase	Thermo Fisher	Cat #11766050
Direct-zol RNA Microprep Kit	Zymo	Cat #R2062
Dual-Luciferase Reporter Assay System	Promega	Cat #E1960

**Deposited data**		

caMPRA and sMPRA data	This paper	GEO: GSE243549
Roadmap Epigenomics data	Kundaje et al.^30^	http://www.roadmapepigenomics.org/
scTHS-seq data	Lake et al.^33^	GEO: GSE97942
JASPAR motif database	Castro-Mondragon et al.^120^	https://jaspar.genereg.net/
DECIPHER consortium	Firth et al.^34^	https://www.deciphergenomics.org/
SFARI database of autism-associated genes	Abrahams et al.^8^	https://gene.sfari.org
Gnomad (pLI and LOEUF scores)	Lek et al.^35^; Karczewski et al.^41^	https://gnomad.broadinstitute.org/
GTEx Consortium	GTEx Consortium^38^	https://gtexportal.org
Eukaryotic Promoter Database	Dreos et al.^121^	https://epd.expasy.org/epd/
HMCA	dbGaP	dbGaP: phs001894.v1.p1
SSC	SFARI	https://www.sfari.org/resource/simons-simplex-collection/

**Experimental models: Cell lines**		

Mouse: Neuro2A	ATCC	Cat #CCL-131
Human: iPSC WTC11 line	Tian et al.^122^	N/A
Experimental models: Organisms/strains		
*Mus musculus*/C57Bl/6J	Cyagen	N/A

**Oligonucleotides**		

CustomArray pool	CustomArray, Bothell, WA	https://www.customarrayinc.com/
sMPRA oligo pool	Twist Biosciences	https://www.twistbioscience.com/
Alt-R CRISPR-Cas9 tracrRNA	IDT	Cat #1072532
Alt-R CRISPR-Cas9 locus targeting crRNA, gctgatggaacaggtaacaa	Osterwalder et al.^123^	N/A
Primers, see Table S5	This paper	N/A
gRNAs, see Table S5	This paper	N/A

**Recombinant DNA**		

pMPRA1	Melnikov et al.^124^	Addgene #49349
pMPRADonor2	Melnikov et al.^124^	Addgene #49353
Firefly luciferase vectors pGL4.12	Promega	Plasmid #E6671
pBA904	Replogle et al.^125^	Addgene #122238
PCR4-Shh::lacZ-H11	Kvon et al.^126^	Addgene # 139098

**Software and algorithms**		

PHAST tools	Siepel et al.^31^	http://compgen.cshl.edu/phast/
BiasAway	Khan et al.^127^	https://biasaway.uio.no/
PWMSCAN	Ambrosini et al.^128^	https://ccg.epfl.ch//pwmtools/pwmscan.php
clusterProfiler	Yu et al.^129^	https://guangchuangyu.github.io/software/clusterProfiler/
DeepSEA	Zhou et al.^46^	http://kipoi.org/models/DeepSEA/beluga/
GREAT	McLean et al.^59^	http://great.stanford.edu/public/html/
MIPgen	Boyle et al.^130^	https://shendurelab.github.io/MIPGEN/
Cutadapt	Martin^131^	https://cutadapt.readthedocs.io/en/stable/
Bwa mem	Li and Durbin^132^	https://bio-bwa.sourceforge.net/bwa.shtml
featureCounts	Liao et al.^133^	https://subread.sourceforge.net/featureCounts.html
UMI-tools	Smith et al.^134^	https://umi-tools.readthedocs.io/en/latest/
Bcftools	Li^135^	https://samtools.github.io/bcftools/bcftools.html
Bamclipper	Au et al.^136^	https://github.com/tommyau/bamclipper
Samtools	Li et al.^137^	http://www.htslib.org/
GERP++	Davydov et al.^138^	http://mendel.stanford.edu/SidowLab/downloads/gerp/index.html
CADD	Rentzsch et al.^139^	http://cadd.gs.washington.edu/
DANN	Quang et al.^140^	https://cbcl.ics.uci.edu/public_data/DANN/
FATHMMnc	Shihab et al.^141^	http://fathmm.biocompute.org.uk/
GATK	Van der Auwera et al.^142^	https://gatk.broadinstitute.org/hc/en-us
Beagle	Browning and Browning^143^	https://faculty.washington.edu/browning/beagle/b4_0.html
motifbreakR	Coetzee et al.^144^	https://github.com/Simon-Coetzee/motifBreakR
STRING	Szklarczyk et al.^145^	https://string-db.org/

### Resource availability

#### Lead contact

• Further information and requests for resources and reagents should be directed to and will be fulfilled by the lead contact, Christopher A. Walsh (christopher.walsh@childrens.harvard.edu).

#### Materials availability

• This study did not generate new unique reagents.

#### Data and code availability

• caMPRA and sMPRA data have been deposited at GEO. Accession numbers are listed in the key resources table. In addition, this paper analyzes publicly available data. These accession numbers for the datasets are listed in the key resources table. Processed data from this paper can be visualized on the UCSC Genome Browser using the following link: http://genome.ucsc.edu/cgi-bin/hgTracks?db=hg38&hubUrl=https://allendiscoverycenter-harhub.s3.us-east-2.amazonaws.com/HAR_hub/hub.txt. All data reported in this paper will be shared by the lead contact upon request.
• This paper does not report original code.
• Any additional information required to reanalyze the data reported in this paper is available from the lead contact upon request.

### Experimental model and subject details

#### Cell lines

Neuro2A (N2A) cells (ATCC, cat #CCL-131) were grown in 10% fetal bovine serum and 1% Penicillin-Streptomycin in Dulbecco’s Modified Eagle Medium with L-Glutamine, 4.5g/L Glucose and Sodium Pyruvate (Fisher, cat #MT10013CV) at 37^o^C. We used a modified version of the male iPSC line WTC11 that contained stably integrated cassettes of a dox-inducible NGN2 and a degron-based inducible dCas9-KRAB^122^. Both cell lines were maintained in a 5% CO_2_ incubator at 37^o^C.

#### Mice

Transient transgenic mice were generated using plasmids containing either the human or chimpanzee versions of HAR3091 or HAR3094 located upstream of a minimal promoter driving a *lacZ* reporter gene. These constructs were generated by Vectorbuilder (VB210119-1206gb, VB210119-1208wrc, VB201008-1098whx, VB201020-1677ynn). Pronuclear injections of these constructs were performed in mice by the Mouse Engineering Core at Dana Farber / Harvard Cancer Center or by Cyagen (Santa Clara, CA). Mouse embryos were harvested at E14.5, bisected, and stained for lacZ expression. Embryos were cleared in 30% sucrose-PBS for imaging. Embryos with successful transgene insertions were determined by PCR for the *lacZ* gene. Embryos were not sexed. All animal experiments conformed to the guidelines approved by the Children’s Hospital Animal Care and Use Committee.

#### Human subjects

From ASD families available through the NIMH repository, we processed 5551 samples (1911 probands) from likely simplex families, and 2277 samples (660 probands) from multiplex families in the Autism Genetic Resource Exchange (AGRE). Variant call format (VCF) files from whole-genome sequencing were obtained for the Homozygosity Mapping Collaborative for Autism (HMCA) from dbGaP phs001894.v1.p1. VCF files for SSC were obtained from SFARI. The number of male and female human subjects in each cohort is indicated in Figure 3A. Research on human samples was conducted with approval of the Committee on Clinical Investigation at Boston Children’s Hospital.

### Method details

#### Selection of HARs, CNEs, VEs

The set of 3171 HARs examined in this study were selected from a number of different studies that identified HARs separately^20^^,^^21^^,^^22^^,^^23^^,^^24^^,^^146^. Identified HARs that overlap were merged.

VEs were selected from active enhancers from the VISTA Enhancer browser^29^. The enhancers were filtered for activity in the brain at E11.5, the time point evaluated by the VISTA enhancer group, and because many VEs are very long (greater than 1kb), VEs were subdivided. Only regions that contained at least mildly species-conserved bases (phastCons > 0.57) based on the 100-way vertebrate alignment from the UCSC Genome Browser^147^ were selected, with regions 50bp or closer merged to form a single element.

CNEs were selected based on epigenetic datasets, species conservation, and population constraint metrics. CNEs were filtered for conserved genomic regions, defined as having a >400 log-odds of being conserved using phastCons with the Viterbi setting^31^. Additionally, CNEs were required to have an enhancer-associated chromatin state (EnhG, Enh, or EnhBiv) based on ChromHMM^30^ in neurospheres, fetal brain, or adult brain. Any elements that were annotated as exonic or splicing were filtered out. Furthermore, no more than 2% of bases in CNEs could have variants in Gnomad^41^.

#### caMPRA design, capture, and construction

Molecular inversion probes were designed to capture ∼500bp regions using the MIPgen program^130^. Repetitive regions were masked prior to the design of targeting arms. Flanking sequence was used to introduce AsiSI, PspXI, and SfiI restriction enzyme sites (NEB, cat #R0630L, R0656L, R0123L), along with a 10bp barcode for each probe. As targeted regions vary in length and many elements are longer than 500bp, probes were designed to double tile the bases of each element. MIPs were synthesized by Customarray, Inc (Redmond, WA). Differences in the number and lengths of captured sequences for HARs, VEs, and CNEs (Figures S4C and S4D) are due to: (1) The capture sequences recommended by MIPgen vary depending on features of the input sequence, including GC content. (2) Because HARs are shorter on average than VEs and CNEs (Figure S4B), we were able to include more sequences per element on the HAR capture panel.

The synthesized MIP oligos were amplified, amplification arms cleaved using MlyI (NEB, cat #R0610L), and purified using Ampure XP beads (Beckman Coulter) at 1.8x volume of the reaction mix. 15ng of the amplified MIP probes were then hybridized to 500ng of DNA from sample NA12878 (Coriell Institute) for 24 hours in 10x Ampligase buffer. The sequences in between the MIP targeting arms were captured by synthesis using Phusion DNA Polymerase (Thermo Fisher, cat #F530L) and circularized using Ampligase (VWR, cat #A3210K) at 60^o^C for 1 hour. Template DNA and uncaptured DNA were degraded using Exonuclease I (Thermo Fisher, cat #EN0582) and Exonuclease III (Thermo Fisher, cat #EN0191) at 37^o^C for 40 minutes and inactivated at 95^o^C for 5 minutes. The captured circles were then amplified using Phusion DNA Polymerase High Fidelity Master Mix (Thermo Fisher, cat #F531L), using primers that added SfiI restriction sites. Amplified, captured sequences were purified using Ampure XP beads at 0.65x to size select and remove unwanted shorter fragments.

The captured sequences and the pMPRA1 construct^124^ (Addgene, cat #49349) were digested with SfiI and ligated using T4 DNA Ligase (Thermo Fisher, cat #EL0014) at 16^o^C overnight. The ligated construct was purified and concentrated using the QIAquick Nucleotide Removal Kit (Qiagen, cat #28306). The ligated constructs were either transformed into 60 vials of Top10 chemically competent cells (Thermo Fisher, cat #C404006) and cultured overnight at 37^o^C in 200ml of LB/Ampicillin, or transformed into 1 vial of MegaX DH10B T1R Electrocompetent Cells (Thermo Fisher, cat #C640003) and plated on LB/Ampicillin agar plates (Molecular Devices X6023 BIOASSAY TRAYS; Fisher Scientific, cat #NC9372402) overnight at 37^o^C. Plasmid DNA was extracted the following day using the Qiagen Plasmid Maxi Kit (Qiagen, cat #12162). This plasmid library containing the captured sequences and a modified pMPRAdonor2^124^ (Addgene, cat #49353) containing an AsiSI site were then digested using AsiSI and PspXI, and the fragment containing the minimal promoter and luciferase gene from pMPRAdonor2 was cloned into the plasmid library containing the captured sequences between the captured element and the barcode. This final construct was then transformed, cultured, and harvested as above.

For the random mutagenesis of HARs, a 25 bp barcode was used. We performed error-prone PCR using the GeneMorph II Random Mutagenesis Kit (Agilent, cat #200550) on the amplified, captured sequences. Based on the error rate of the Mutazyme II polymerase and PCR yield, we performed 7 cycles of error-prone PCR with 20ng of input captured sequence, with 25bp random barcode reverse primer. These mutagenized sequences were then cloned into the modified pMPRA1 construct as described above. In order to associate the mutagenized sequence with the random barcode, the cloned plasmid library containing the captured sequences was PCR amplified with primers containing sequencing adapters and sent out for 2x250bp sequencing on HiSeq Instruments at Psomagen (Rockville, MD).

#### sMPRA design and construction

An oligo pool containing 238bp sequences centered on each of the 1,693 rare, recessive variants identified in HARs, VEs, or CNEs in the HMCA cohort and in HARs or VEs in the NIMH cohort with or without the variant of interest was synthesized by Twist Biosciences. Each sequence was represented 10 times in the oligo pool with different 12-mer barcodes. The oligo pool was amplified with PCR primers to add SfiI restriction sites, and then cloned as described for caMPRA.

#### Cell culture and transfection for caMPRA and sMPRA

N2A cells (ATCC, cat #CCL-131) were cultured in DMEM with L-Glutamine, 4.5g/L Glucose and Sodium Pyruvate (Fisher, cat #MT10013CV) with 10% fetal bovine serum and 1% penicillin/streptavidin at 37^o^C. Cells were maintained in 10 cm TC-treated plates and split 1:5 every 4 days or when confluent with 0.25% (w/v) Trypsin – 0.53mM EDTA (Fisher, cat #25200-114). To minimize confounding due to passage number, we limited passage numbers to P3-6. For transfections, N2A cells were transfected at 70% confluency using Lipofectamine LTX with PLUS reagent (Thermo Fisher, cat #15338100) with 15ug of caMPRA plasmid, and cells were incubated with the transfection mix for 24 hours. After 1 day, media was changed. Cells were harvested either 1 day or 3 days after transfection. Cell pellets were washed with 1x PBS, and mRNA was extracted using the Dynabead mRNA Direct kit (Thermo Fisher, cat #61012), according to the manufacturer’s instructions. mRNA was reverse transcribed using Superscript VILO Master Mix with EZ DNase (Thermo Fisher, cat #11766050). caMPRA barcodes were extracted using PCR amplification with primers containing Illumina adapters for both the cDNA and plasmid pools and sent out for 150bp sequencing using Hiseq instruments at Psomagen (Rockville, MD).

#### Targeted sequencing of NIMH cohort

From the ASD families available through the NIMH repository, we processed 5551 samples (1911 probands) from likely simplex families, and 2277 samples (660 probands) from multiplex families in the Autism Genetic Resource Exchange (AGRE). The likely simplex families include families with one affected proband and no siblings and families with one affected proband and one or more unaffected siblings. Molecular inversion probes (MIPs) were designed, synthesized, and amplified as described above with the following changes: MIPs were designed to capture ∼240 bp regions and were hybridized to a pool of DNA from NIMH samples. The purified library was quantified using a tapestation and sequenced at 2x150bp by Psomagen (Rockville, MD).

#### LacZ reporter assay (random integration)

We cloned either the human or chimpanzee versions of HAR3091 or HAR3094 upstream of a minimal promoter driving a *lacZ* reporter gene with Vectorbuilder (VB210119-1206gb, VB210119-1208wrc, VB201008-1098whx, VB201020-1677ynn). Pronuclear injections of these constructs were performed in mice by the Mouse Engineering Core at Dana Farber / Harvard Cancer Center or by Cyagen (Santa Clara, CA). Mouse embryos were harvested at E14.5, bisected, and stained for lacZ expression. Embryos were cleared in 30% sucrose-PBS for imaging. Embryos with successful transgene insertions were determined by PCR for the lacZ gene. Because these mice are analyzed at F0, lacZ expression is dependent on the distribution and number of cells that integrate the reporter construct and the genomic location of the integration. Consequently, we expect that expression patterns driven by the sequence of interest rather than by the integration site will be consistently observed in multiple embryos, and we examined at least 10 PCR-positive embryos per construct to account for this variability. E14.5 embryos have an average crown-rump length of 12mm.

#### LacZ reporter assay (site-specific integration)

Transgenic E11.5 mouse embryos were generated as described previously^123^. Briefly, super-ovulating female FVB mice were mated with FVB males and fertilized embryos were collected from the oviducts. Regulatory element sequences were synthesized by Twist Biosciences. Inserts generated in this way were cloned into the donor plasmid containing minimal Shh promoter, lacZ reporter gene and H11 locus homology arms (Addgene, cat #139098) using NEBuilder HiFi DNA Assembly Mix (NEB, cat #E2621). The sequence identity of donor plasmids was verified using long-read sequencing (Primordium). Plasmids are available upon request. A mixture of Cas9 protein (Alt-R SpCas9 Nuclease V3, IDT, cat #1081058, final concentration 20 ng/μL), hybridized sgRNA against H11 locus (Alt-R CRISPR-Cas9 tracrRNA, IDT, cat #1072532 and Alt-R CRISPR-Cas9 locus targeting crRNA, gctgatggaacaggtaacaa, total final concentration 50 ng/μL) and donor plasmid (12.5 ng/μL) was injected into the pronucleus of donor FVB embryos. The efficiency of targeting and the gRNA selection process is described in detail in^123^. Embryos were cultured in M16 with amino acids at 37^o^C, 5% CO_2_ for 2 hours and implanted into pseudopregnant CD-1 mice. Embryos were collected at E11.5 for lacZ staining as described previously^123^. Briefly, embryos were dissected from the uterine horns, washed in cold PBS, fixed in 4% PFA for 30 min and washed three times in embryo wash buffer (2 mM MgCl_2_, 0.02% NP-40, and 0.01% deoxycholate in PBS at pH 7.3). They were subsequently stained overnight at room temperature in X-gal stain (4 mM potassium ferricyanide, 4 mM potassium ferrocyanide, 1 mg/mL X-gal and 20 mM Tris pH 7.5 in embryo wash buffer). PCR using genomic DNA extracted from embryonic sacs digested with DirectPCR Lysis Reagent (Viagen, cat #301-C) containing Proteinase K (final concentration 6 U/mL) was used to confirm integration at the H11 locus and test for presence of tandem insertions (see ^123^ for details). Only embryos with donor plasmid insertion at H11 were used. The stained transgenic embryos were washed three times in PBS and imaged from both sides using a Leica MZ16 microscope and Leica DFC420 digital camera. E11.5 embryos have an average crown-rump length of 6mm.

#### CRISPR inhibition in iPSC-derived neurons

Guide RNAs (gRNAs) were designed to target the middle 200bp interval of HAR3091 and HAR3094 using GuideScan^148^ with a specificity score cut-off > 0.2. Non-targeting control (NTC) gRNAs and gRNAs targeting the *IL1RAPL1* transcription start site (TSS) are from^149^. gRNA sequences can be found in Table S5. gRNAs were cloned into pBA904 (RRID: Addgene_122238), as previously described^125^. Lentivirus was made for each gRNA by ultracentrifugation.

We used a modified version of the iPSC line WTC11 that contained stably integrated cassettes of a dox-inducible NGN2 and a degron-based inducible dCas9-KRAB^122^. This modified WTC11 line was differentiated into neurons by inducing NGN2 expression as previously described^150^. Twice the suggested number of cells (e.g. 2x10^5^ cells per well in a 24-well plate) were plated at D0 to account for incomplete lentiviral infection, and immediately after plating, lentivirus was added at MOI 0.7 (so that ∼50% of cells would be infected with lentivirus). dCas9-KRAB expression was induced by the addition of 20uM trimethoprim (Sigma Aldrich, cat #92131) from D0 until the neurons were collected at D18, and 1 ug/ml puromycin was added from D2-D7 to select for infected neurons. RNA was extracted at D18 using the Direct-zol RNA Microprep Kit (Zymo, cat #R2062) per the manufacturer’s instructions, and cDNA was synthesized from RNA with the SuperScript VILO cDNA Synthesis Kit (Thermo Fisher, cat #11756050). RT-qPCR was performed using Brilliant II SYBR Green Low ROX qPCR Master Mix (Agilent, cat #600830) on a CFX384 Touch Real-Time PCR Detection System (BioRad) for *IL1RAPL1* and *GAPDH* (primer sequences in Table S5) in triplicate.

#### Luciferase assays

Wild-type (WT) and mutant sequences of HAR3091 and HAR3094 were generated through PCR amplification from Promega Control Male human DNA (Promega, cat #G1471) and proband genomic DNA using primers containing unique restriction sites for directional cloning into the minimal promoter pGL4.25 luciferase vector. Two families harboring the same variant in HAR3091 (chrX:29275880, G>T) were amplified independently and cloned into separate plasmids containing the same variant. Plasmids containing WT or patient variant sequences of HAR3091 and HAR3094 were transformed into Top10 chemically competent cells (Thermo Fisher, cat #C404006). Genotypes and structures of the final plasmids were confirmed using Sanger sequencing. Plasmids (75ng) were co-transfected along with control Renilla (25ng) into mouse neuroblastoma Neuro-2a cells (N2a) (ATCC, cat #CCL-131) in 96-well plates using Lipofectamine 3000 (Thermo Fisher, cat #L3000015). Luciferase activity was assessed 48 hours post-transfection using the dual-luciferase reporter assay system (Promega, cat #E1960). Firefly luciferase activity was normalized to Renilla luciferase activity for each well of the 96-well plates. The luciferase activity measurements were performed with 8 replicates. We tested each cloned plasmid containing WT or patient variant sequences for HAR3091 and HAR3094, as well as the empty vector. Data from the two plasmids generated from different families with the same variant in HAR3091 yielded similar results, and are shown as one variant (Figures 4D and S17).

### Quantification and statistical analysis

#### Assessing epigenomic annotations in human tissue

ChromHMM annotations from the Epigenomics Roadmap Project^30^ were overlapped with HARs, CNEs, and VEs using bedtools intersect^151^ to identify the number of elements in each class that were annotated as active (TssA, TssAFlnk, TxFlnk, Tx, TxWk, EnhG, Enh, TssBiv, BivFlnk, or EnhBiv in the 15-state model) in each assessed tissue. For cell type-specific annotations in the adult brain, scTHS-seq data was used^33^ to determine accessibility in different brain cell types for HARs, VEs, and CNEs.

#### TF binding analysis

We examined HARs, VEs, and CNEs, as well as matched background sequences generated using BiasAway^127^, for transcription factor binding sites. PWMSCAN^128^ was used to scan sequences for motifs from the JASPAR motif database^120^ to identify potential transcription factor binding sites. To control for false positives, a p-value cutoff of 10^-4^ was used. The presence of motifs was then aggregated and the enrichment of specific motifs in HARs, VEs, or CNEs compared to matched background sequences was determined. P-values for enrichment were generated using the hypergeometric test and were adjusted for multiple hypothesis testing with the Benjamini-Hochberg correction. Gene set enrichment analysis for TFs with motifs in HARs, VEs, and CNEs was performed using clusterProfiler^129^ and adjusted for multiple hypothesis testing with the Benjamini-Hochberg correction.

In addition to motif-based matching, HARs, VEs, and CNEs were also annotated using DeepSEA^46^ using the online DeepSEA server. We used the Beluga model that was trained on 2,002 chromatin features. TF ChIP-seq features were selected for, and only those with an e-value (defined as the expected proportion of common SNPs with a larger predicted effect) of less than 0.05 were interpreted as associated with the element.

#### Analysis of genes near HARs, VEs, and CNEs

Gene ontology analysis was performed with GREAT^59^ with the binomial test at 5% FDR. The binomial test at 5% FDR were also used to assess whether HARs, VEs, and CNEs are enriched near disease-associated genes. HARs, VEs, and CNEs were assigned to nearby genes as previously described^59^. We separated genes implicated in severe, developmental disorders from the DECIPHER consortium (v. 13_7_2022)^34^ based on the phenotypes of the affected individuals. If affected individuals had phenotypes in multiple body systems, affected genes were assigned to all affected body systems. We also examined autism-associated genes from the SFARI database^8^ and genes specifically expressed in the brain using bulk RNA sequencing data from the GTEx consortium^38^. We defined brain-specific genes as those only expressed in brain tissues (median TPM > 25).

To examine whether autism-associated genes and genes near HARs, VEs, or CNEs were enriched for dosage-sensitive genes, we examined pLI and LOEUF scores^35^^,^^41^. pLI > 0.9 and low LOEUF scores indicate loss-of-function intolerance. The hypergeometric test was used to test whether ASD-associated genes from the SFARI database and genes near HARs, VEs, or CNEs were enriched for genes with pLI > 0.9 at 5% FDR. The Wilcoxon rank-sum test was used to test whether the LOEUF scores of ASD-associated genes or genes near HARs, VEs, or CNEs differed from LOEUF scores for all genes at 5% FDR.

To assess whether variants enriched in cases compared to controls are enriched near genes that perform specific functions, we examined rare, recessive variants in HARs, VEs, or CNEs in the HMCA cohort and HARs or VEs in the NIMH cohort that were found in an excess of ASD cases compared to controls using GREAT. As the background set, we used all rare, recessive variants in HARs, VEs, or CNEs in the HMCA cohort and HARs or VEs in the NIMH cohort.

#### MPRA analysis

To count barcodes to assess regulatory activity, sequencing data from the plasmid DNA and cDNA libraries described above were processed with cutadapt to remove adapters^131^. Barcodes were extracted using UMI-tools^134^ and reads were mapped using bwa mem^132^. Reads were assigned to caMPRA probes using featureCounts^133^. The 10bp barcode for caMPRA or the 12bp barcode for sMPRA were clustered to recover barcodes with small sequencing errors using the multiplexed version of the UMI-tools directional method.

Barcodes for plasmid and cDNA samples were normalized to a barcode per million format to remove bias due to sequencing coverage. Each cDNA barcode was normalized to the barcode count in the plasmid pool, and log_2_ transformed. Only barcodes that were found in the plasmid pool and all cDNA replicate pools (5 for caMPRA and 6 for sMPRA) were used in the analysis. Statistical significance was assessed with the Wilcoxon test at 5% FDR.

For caMPRA, we were able to examine 2932 HARs, 1702 VEs, and 5155 CNEs after filtering. Elements were considered active if at least one probe overlapping that element was active in the assay because the functional components of a given element may only have been captured in a single probe. Note that prior studies observed limited concordance in enhancer activity between different portions of the same HAR in MPRA assays^104^^,^^105^. The proportion of active probes (sequences) is shown in Figure S6B, and full results are detailed in Table S2.

For sMPRA, we were able to assess 1016 variants after filtering. Variants were considered to modulate enhancer activity if the sequence containing the variant site (with or without the variant) had significant enhancer activity, and there was a significant difference between a sequence with and without the variant. The results for the 141 variants contained within sequences with significant enhancer activity can be found in Table S4. Note that this filtering excluded HAR3091 and HAR3094, which contained sequences that were active by caMPRA but not sMPRA, suggesting that long sequence contexts are required for enhancer activity in these elements. This filtering also excluded VE235/hs1066.1 and VE854/hs576, which were not active by caMPRA or sMPRA despite strong enhancer activity *in vivo* (Figure 5), suggesting that their activity likely requires TF repertoires that are not present in N2A cells.

#### Analysis of caMPRA data from random mutagenesis

Analysis of the plasmid and cDNA barcode pools was performed as described above. Variants from each caMPRA probe were called using bcftools mpileup^135^, and associated with the appropriate barcode. Sequences captured using MIPs may include regions that flank the sequence of interest. Mutagenized sequences that only included variants in the flanking sequence were excluded. Variants found in NA12878 compared to the reference genome were filtered out. The correlation between replicate experiments (Figure S8) was assessed prior to removing mutagenized sequences that only included variants in the flanking sequence.

#### MIP sequencing, processing, and variant calling

Analysis of targeted sequencing was performed using a custom pre-processing pipeline combined with GATK-based variant calling. Briefly, sequenced reads were trimmed for adapters using cutadapt^131^. UMI-tools^134^ was used to extract the 5bp unique molecular index (UMI), and reads were mapped to the human genome (hg19) using bwa mem^132^. Reads that mapped off-target compared to the intended target were filtered out. The extension and ligation arms (the targeting arms) were clipped off the mapped reads using bamclipper^136^. Samtools was used to remove multimapping reads, unpaired/broken read pairs, and unmapped reads^137^. UMI-tools was used to collapse sequences based on UMI sequences. Finally, sequences were base recalibrated using GATK base recalibrator, and variants were joint-called using the GATK Haplotype caller and suggested GATK best practices, including Variant Quality Score Recalibration (VQSR)^142^. After targeted sequencing and processing, we were able to resolve HARs in 6464 individuals, VEs in 5273 individuals, and CNEs in 5983 individuals. Available data from the HMCA and SSC cohorts were previously joint-called using GATK best practices and VQSR^6^^,^^11^. Compound heterozygous calls were made using Beagle 4^143^, which has improved phasing with pedigrees that include family information. Chromosomal multisample VCFs were phased using beagle.r1399 with the following settings: impute=false window=5000 overlap=500, and the family pedigree file. This is similar to methodology used in a prior publication that was shown to work well for protein-coding changes^6^. However, we acknowledge that phasing rare events, particularly if the parent genotype is missing or low quality is challenging and that phasing accuracy is not as high for rare variants as it is for common variants.

#### Variant filtering, classification, and analysis

The HMCA consanguineous cohort was filtered for variants of AD > 2, DP > 10, and GQ > 20. For the targeted sequencing of the NIMH cohort, variants were required to have a minimum of 10x coverage, GQ > 20, and AD > 4. Only variants produced from collapsed reads were used for accuracy. The Simon Simplex Collection (SSC) cohort of 8,186 individuals was filtered to remove alleles with AD <3, DP<5, and GQ <20, and those not meeting the PASS filter. To ensure the accuracy of the data harmonization, the rates of likely neutral variants (rare variants at non-conserved sites) were compared between cases and controls for HARs, VEs, and CNEs in all cohorts, as well as total rates of homozygous and heterozygous events.

For recessive variant analysis, our definition included homozygous, compound heterozygous, and hemizygous variants (specifically in male individuals for the X chromosome). Because hemizygous variants are much more likely to appear, we performed our analysis with each sex separately when examining genome-wide rates.

In order to enrich for functionality, we created a classification that uses an ensemble of different conservation-based variant effect predictors – GERP++^138^, CADD^139^, DANN^140^, FATHMMnc^141^ – to annotate variants and base positions. Variants were filtered to exclude those within exonic regions of protein-coding genes (based on RefSeq and Gencode v28). For variants that fall within either the UTRs, within 1 kb upstream of a transcriptional start site, or within a predicted promoter element from the Eukaryotic Promoter Database^121^, these variants must overlap a conserved element from the 100-way phastCons from the UCSC genome browser. All variants were filtered for GERP > 2 and (CADD > 15 or DANN > 0.85 or FATHMMnc > 0.85).

The variant contributions of rare germline events were assessed for rare, recessive and *de novo* predicted damaging variants identified in individuals with ASD and healthy familial controls. Statistical testing of variant contributions was performed as follows. First, the odds ratio (OR), standard error, and 95% confidence intervals were calculated using the following approach^49^: OR = (a/b)/(c/d), where a = number of cases with variants, b = number of cases without variants, c = number of controls with variants, and d = number of controls without variants. The standard errors of the log odds ratio were calculated using the following formula: $SE\left(\ln\left(OR\right)\right)=\sqrt{\frac{1}{a}+\frac{1}{b}+\frac{1}{c}+\frac{1}{d}}$. The 95% confidence intervals were determined using 95%CI=exp(ln(OR)±1.96xSE(ln(OR))). *p*-values of the ORs were calculated under the assumption of the deviation from a normal distribution, using: $z=\frac{\ln\left(OR\right)}{SE\left(\ln\left(OR\right)\right)}$. The allele frequency (AF) cut-off for statistical analysis was set at either AF < 0.005 (HMCA and SSC) or AF < 0.001 (NIMH), using the lowest AF where there were >5 predicted damaging variants for cases and controls for each sex. The contribution of variants to ASD risk was estimated as the difference between the rate of rare, recessive variants in cases compared to controls as previously described^6^. Significance was assessed at 5% FDR.

We compared the predicted damaging variant rates in cases and controls, against those of neutral events, which were defined as variants at non-conserved nucleotide positions with no predicted functional effect. As expected, significant excesses of neutral variants were not detected in the NIMH and SSC cohorts. However, given the known consanguinity in the HMCA cohort, the rates of neutral events were elevated in cases vs controls in this cohort, as previously shown for synonymous variants^6^. Therefore, in cohorts with known elevations of homozygosity that could impact the recessive contribution (e.g., HMCA), we determined the rates of likely benign events at non-conserved sites within gene promoters that have no predicted functional impact under the assumption that these rates should be equivalent in cases and controls due to the lack of selection bias on the sites. Next, the rates of predicted damaging events in cases were reduced proportionally to the excess detected in the non-conserved sites, as was done previously using synonymous rates for recessive protein-coding variation^6^. Following correction for elevated consanguinity, variation contributions and significance were determined, using the above described approach.

Variants were analyzed for potential effects on transcription factor binding sites using motifbreakR^144^ on PWMs from JASPAR 2022 retrieved using MotifDb^120^. We used method=”log” and threshold=1e-4 with motifbreakR and only reported predictions annotated as having a “strong” effect.

#### Protein-protein interaction networks

Variants found in HARs, VEs, or CNEs in HMCA and variants found in HARs or VEs in NIMH were aggregated to the level of individual HARs, VEs, and CNEs. HARs, VEs, and CNEs with a numerical excess of variants found in patients compared to controls were associated with nearby genes using GREAT^59^. A protein-protein interaction network for these nearby genes was constructed using STRING version 11.5 using the online interface with default parameters^145^.

To examine whether proteins encoded by genes near variants found in a numerical excess of patients compared to controls had more interactions than expected, we used the STRING online interface to determine the PPI enrichment p-value. All proteins encoded by genes near variants detected in HARs, VEs, or CNEs in the HMCA cohort or HARs or VEs in the NIMH cohort were used as the background set.

#### Analysis of CRISPRi and luciferase assays

To analyze the CRISPR inhibition (CRISPRi) data, the quantity of *IL1RAPL1* for each sample was calculated by comparing the Ct value for *IL1RAPL1* to a standard curve of pooled samples and then normalized to the expression of the housekeeping gene *GAPDH* in the same sample. This normalized value was then divided by the normalized quantity of *IL1RAPL1* in samples infected with NTC gRNAs. Each point represented in Figures 4 and S16 is from a separate well; each condition was tested in at least 3 different differentiations. The Wilcoxon rank-sum test was used to compare each individual gRNA to the NTC gRNAs, and p-values for gRNAs targeting the same region were combined with Fisher’s method. P-values were adjusted with the Benjamini-Hochberg correction and considered significant at 5% FDR.

For the luciferase assays, the Wilcoxon rank-sum test was used to compare each test sequence to the control sequence, and p-values for each replicate were combined with Fisher’s method. P-values were adjusted with the Benjamini-Hochberg correction and considered significant at 5% FDR. Note that the plasmid containing WT HAR3094 had increased enhancer activity compared to the empty vector, whereas there was no difference between the plasmid containing WT HAR3091 and the empty vector.

## Supplemental information

Document S1. Figures S1–S22, Table S5, and Data S1

Table S1. Coordinates (hg19), TF motif enrichments, and GREAT gene ontology results of HARs, VEs, and CNEs, related to Figure 1 The *p* values for TF motif enrichment were calculated with the hypergeometric test and adjusted with the Benjamini-Hochberg correction. See STAR Methods for full details.

Table S2. caMPRA results for HARs, VEs, and CNEs, related to Figure 2 The *p* values were calculated with the Wilcoxon test and adjusted with the Benjamini-Hochberg correction. Full details of statistical analyses are in STAR Methods.

Table S3. caMPRA results from random mutagenesis of HARs, related to Figure 2 The *p* values were calculated with the Wilcoxon test and adjusted with the Benjamini-Hochberg correction. Full details of statistical analyses are in STAR Methods.

Table S4. Rare, recessive variants at conserved bases in ASD cohorts, related to Figure 3

Document S2. Article plus supplemental information
