# Supplementary material for: Rare variation in non-coding regions with evolutionary signatures contributes to autism spectrum disorder risk
Source: Cell Genom. 2024 Jul 16;4(8):100609. doi: 10.1016/j.xgen.2024.100609 (PMC11406188; doi:10.1016/j.xgen.2024.100609)
Supplement: Document S1. Figures S1–S22, Table S5, and Data S1 [file mmc1.pdf]

Cell Genomics, Volume *4*

Supplemental information

Rare variation in non-coding regions with evolutionary signatures contributes to autism spectrum disorder risk

Taehwan Shin, Janet H.T. Song, Michael Kosicki, Connor Kenny, Samantha G. Beck, Lily Kelley, Irene Antony, Xuyu Qian, Julieta Bonacina, Frances Papandile, Dilenny Gonzalez, Julia Scotellaro, Evan M. Bushinsky, Rebecca E. Andersen, Eduardo Maury, Len A. Pennacchio, Ryan N. Doan, and Christopher A. Walsh

Data S1: Feasibility of assessing patient variants in HAR3091 and HAR3094 using *in vivo* enhancer reporter assays, Related to Fig. 4.

To assess the human and chimp versions of HAR3091 and HAR3094, we used a transient transgenic mouse assay where reporter constructs containing test regions upstream of the hsp68 promoter and the lacZ gene were randomly integrated (Fig. 4B). With random integration, reporter expression is dependent on the genomic location of the integration, and a large number of embryos need to be assessed for each construct in order to confidently identify expression patterns driven by the sequence of interest rather than by the integration site. By examining 10+ PCR-positive embryos per reporter construct, we were able to identify differences in the enhancer activity of HAR3091 and HAR3094. However, the large variability between embryos (Fig. S15) made it clear that this methodology would not provide us with the confidence needed to assess less overt differences, such as those caused by ASD patient variants.

Dr. Len Pennacchio's lab previously developed a method to reduce variability through targeted integration of an enhancer reporter construct containing the Shh promoter into the H11 locus ([S1]). His lab has also contributed extensively to the Vista Enhancer Browser ([S2]), which has tested >3000 non-coding elements for enhancer activity, primarily with the same random integration method we used to assess HAR3091 and HAR3094. Although many of these elements replicate their enhancer activity with the targeted integration method, some elements do not drive enhancer activity in the H11/Shh context likely due to differences between the Shh promoter and the hsp68 promoter. Unfortunately, HAR3091 and HAR3094 fell into this category and did not drive enhancer activity with the H11/Shh system, making it infeasible to assess the function of the ASD patient variants with the targeted integration approach. This motivated us to instead test HAR3091 and HAR3094 in *in vitro* luciferase assays, where we observe that ASD patient variants can alter enhancer activity (Fig. 4D). We were able to use the targeted integration approach to assess patient variants in VE235/hs1066.1 and VE854/hs576 *in vivo* (Fig. 5).

Supplemental Figures

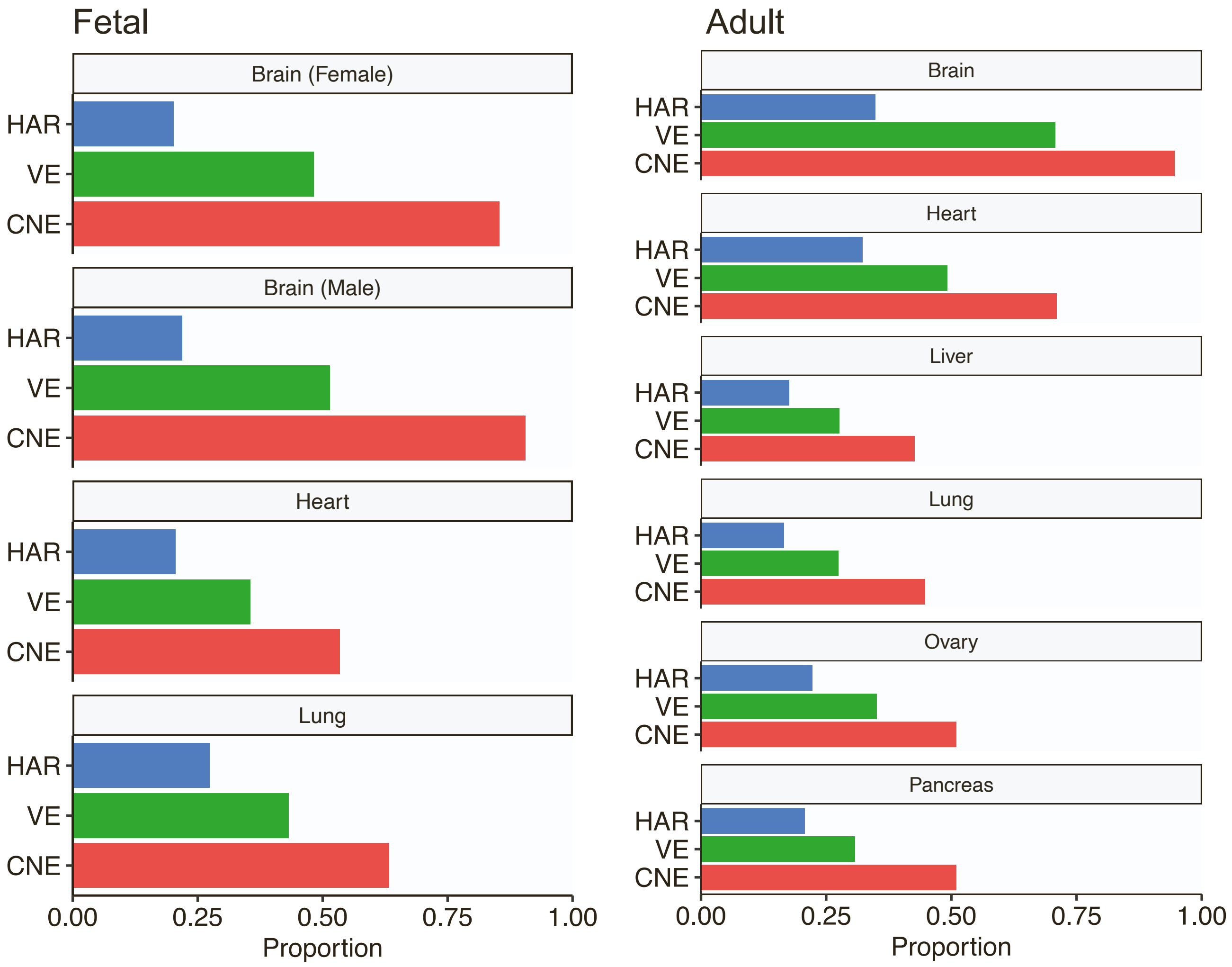

Figure S1: **Proportion of HARs, VEs, and CNEs predicted to be active in fetal (left) and adult (right) tissue by ChromHMM from the Roadmap Epigenomics Project ([S3]) (STAR Methods), Related to Fig. 1.**

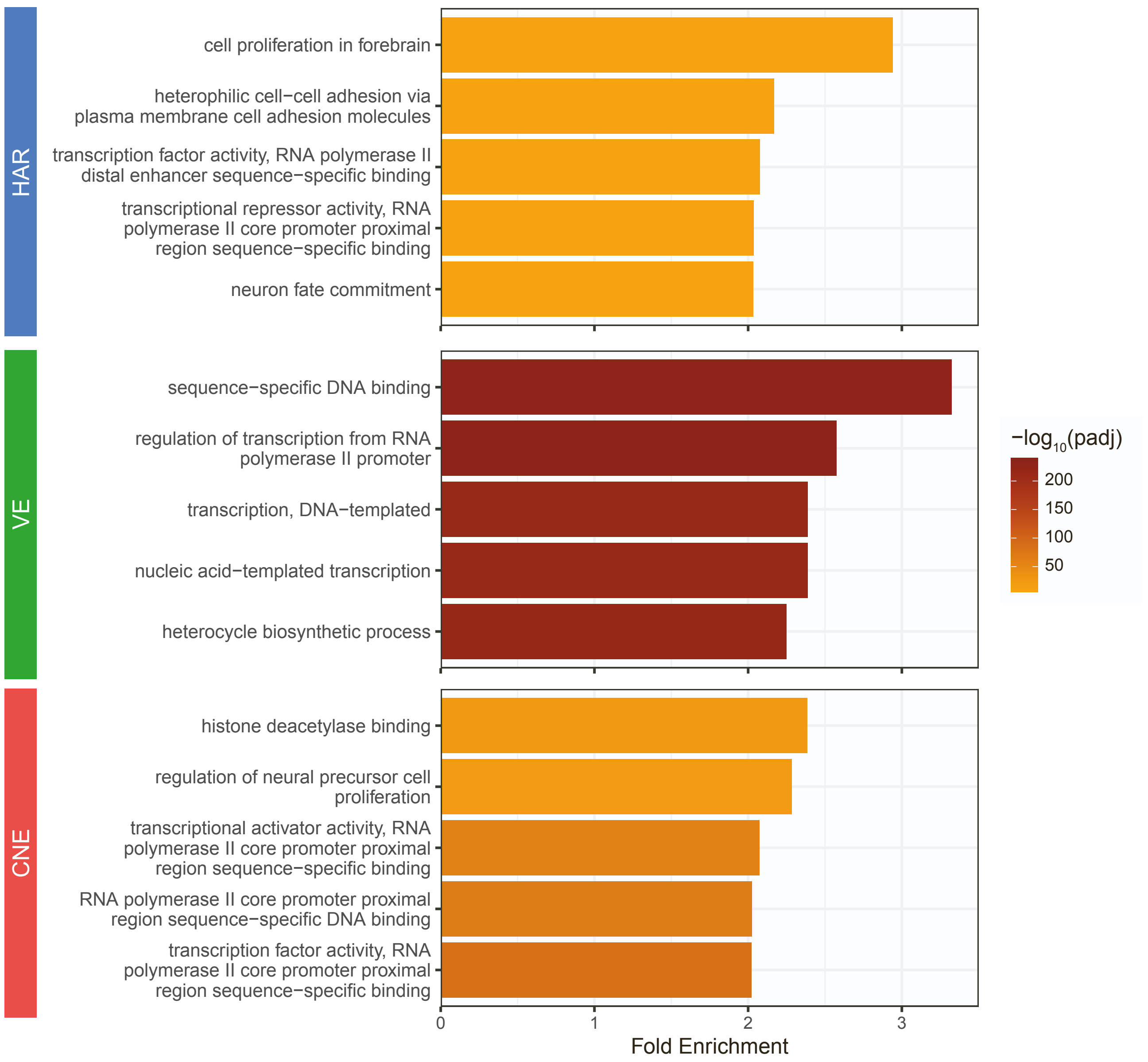

Figure S2: **Gene ontology enrichments for genes near HARs, VEs, and CNEs using GREAT ([S4]), Related to Fig. 1.** The top five enriched terms from the binomial test are plotted.

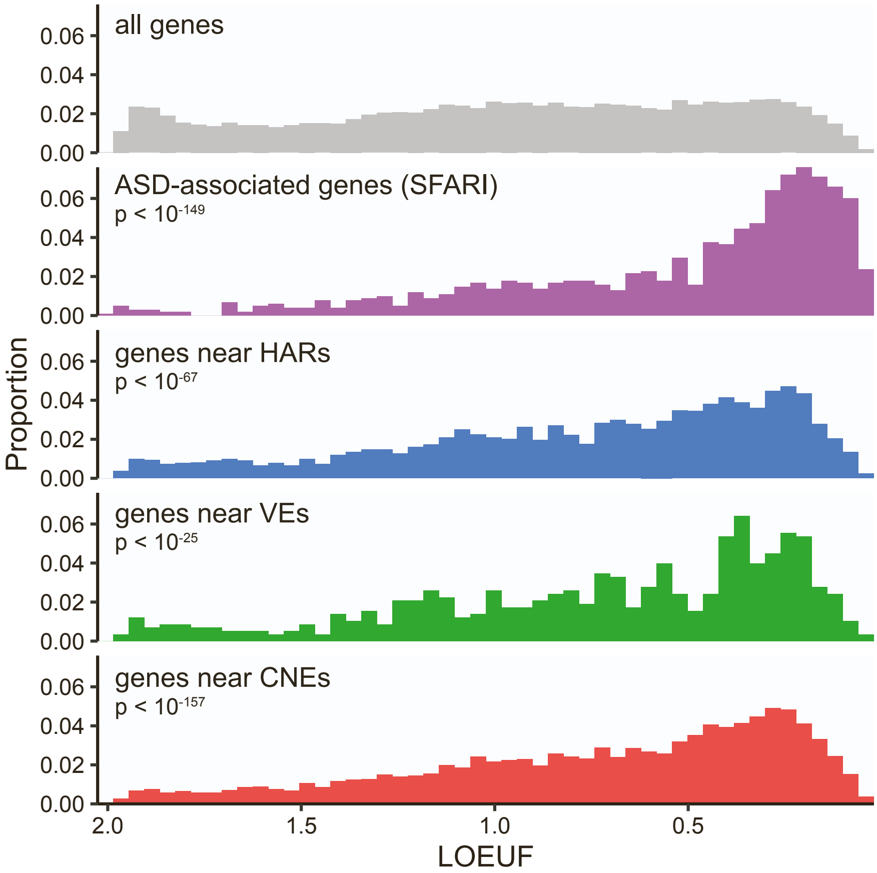

Figure S3: **ASD-associated genes from the SFARI database ([S5]) and genes near HARs, VEs, and CNEs are enriched for low LOEUF scores compared to all genes, Related to Fig. 1.** Genes that are loss-of-function intolerant have low LOEUF scores ([S6]).

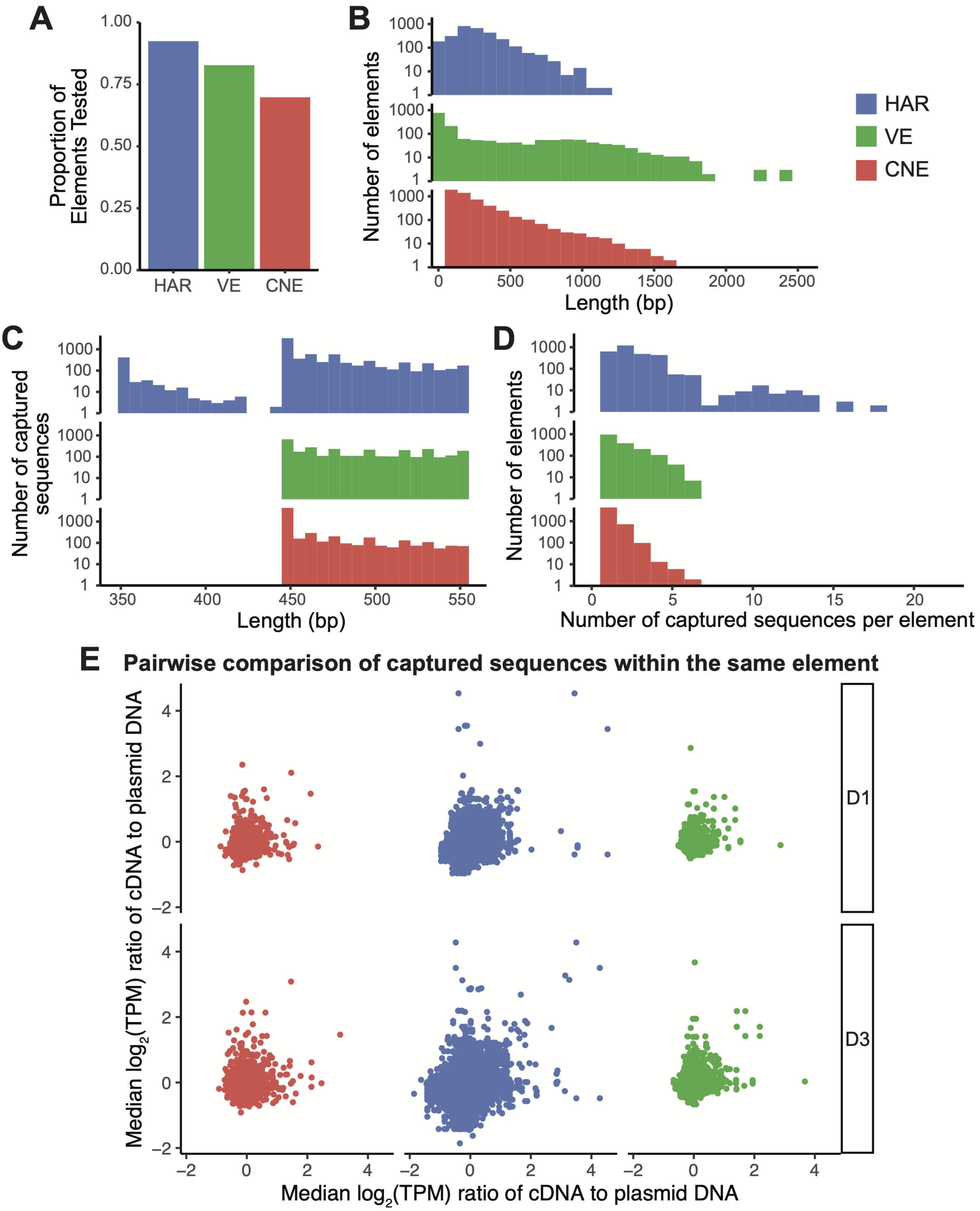

Figure S4: **Features of caMPRA for HARs, VEs, and CNEs, Related to Fig. 2.** (A) Proportion of HARs, VEs, and CNEs tested in caMPRA. (B) Distribution of the length of HARs, VEs, and CNEs. (C) Length and number of sequences captured for HARs, VEs, and CNEs. (D) Distribution of the number of captured sequences per HAR, VE, and CNE. (E) Normalized ratio of cDNA to plasmid DNA (enhancer activity) for sequences captured from the same HAR, VE, or CNE. The enhancer activity of captured sequences from the same element is not correlated.

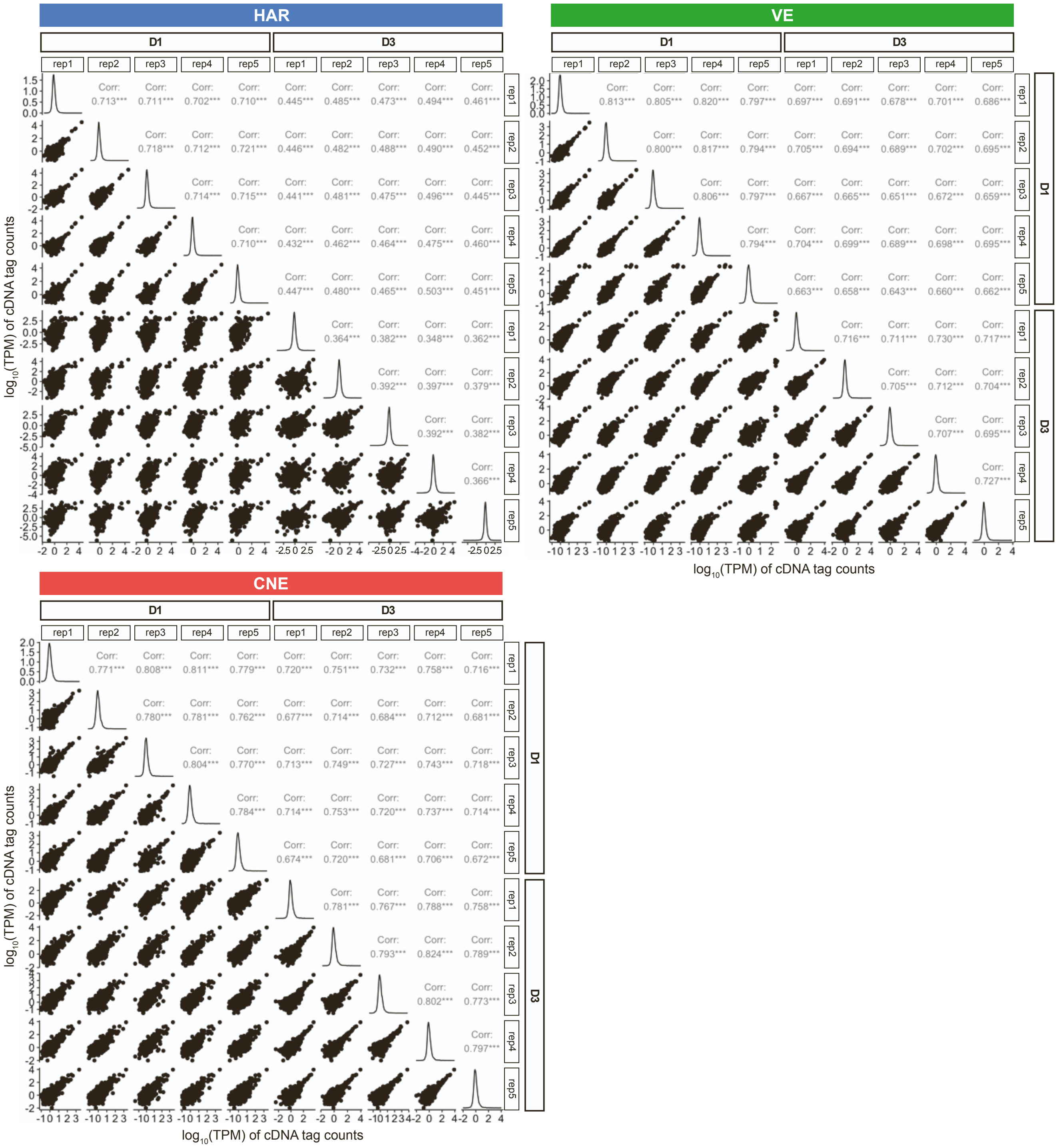

Figure S5: **Enhancer activity in caMPRA of HARs, VEs, and CNEs is well-correlated between replicates and between collection timepoints, Related to Fig. 2.**

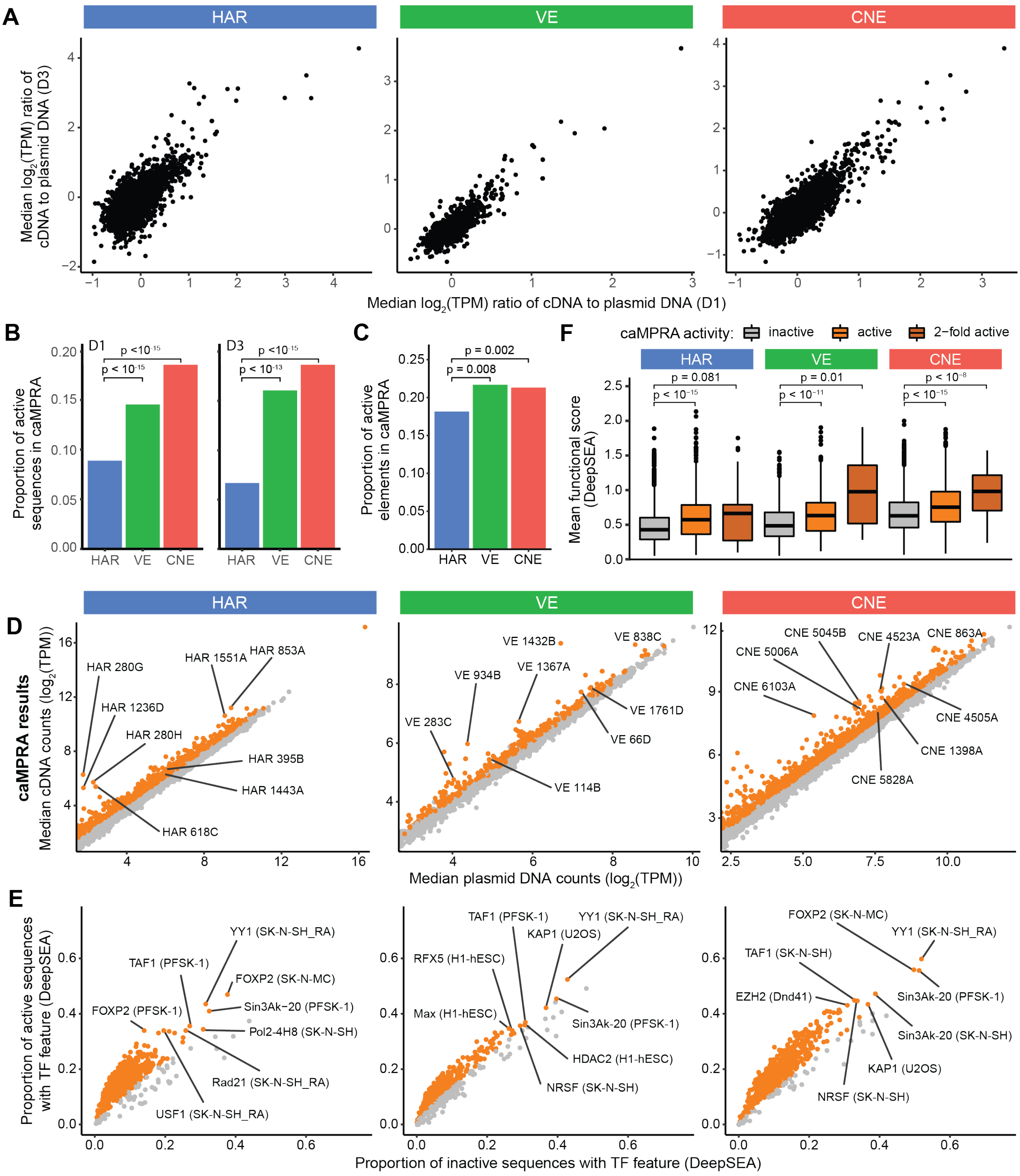

Figure S6: **HARs, VEs, and CNEs display enhancer activity in a capture-based Massively Parallel Reporter Assay (caMPRA), Related to Fig. 2 and STAR Methods.** (A) The enhancer activity of captured sequences between the D1 and D3 caMPRA experiments is highly correlated. (B) Proportion of captured sequences that are active in caMPRA for HARs, VEs, and CNEs when assessed one (D1) or three (D3) days after transfection. Fig. 2 shows the results from the D3 caMPRA experiment. (C) Proportion of HARs that have enhancer activity in at least one captured sequence in the D1 caMPRA is significantly lower than VEs or CNEs by the chi-square test after FDR correction. (D) Normalized cDNA counts vs normalized plasmid counts for sequences captured from HARs, VEs, and CNEs from the D1 caMPRA experiment. Sequences with significant enhancer activity are in orange. (E) TF features were predicted by DeepSEA ([S7]) for each captured sequence. TF features significantly enriched in active sequences in the D1 caMPRA experiment are shown in orange. Representative TF features are marked in the format: TF (cell type). (F) Sequences captured from HARs, VEs, and CNEs are classified as inactive, active, or 2-fold active from the D1 caMPRA experiment and compared to their mean functional score from DeepSEA (average of $-log_{10}(e - value)$ for every feature) ([S7]). The level of activity in caMPRA is correlated with the predicted mean functional score from DeepSEA. P-values were determined with the hypergeometric test and adjusted by FDR correction.

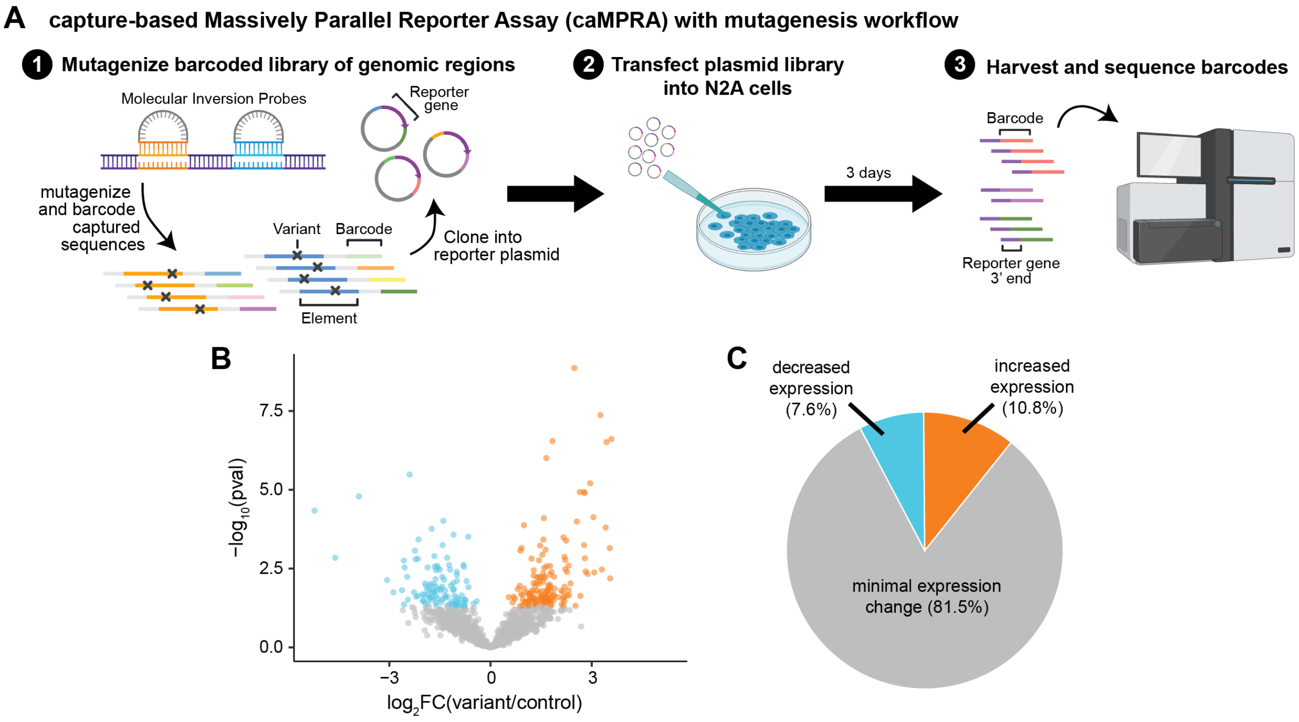

Figure S7: **Random variants in HARs can modulate enhancer activity, Related to Fig. 2.** (A) Schematic of caMPRA with random mutagenesis. (B) Volcano plot of fold change in expression and adjusted p-value for each mutagenized sequence. (C) Pie chart of percent of mutagenized sequences with decreased expression, increased expression, or no statistically significant change in expression.

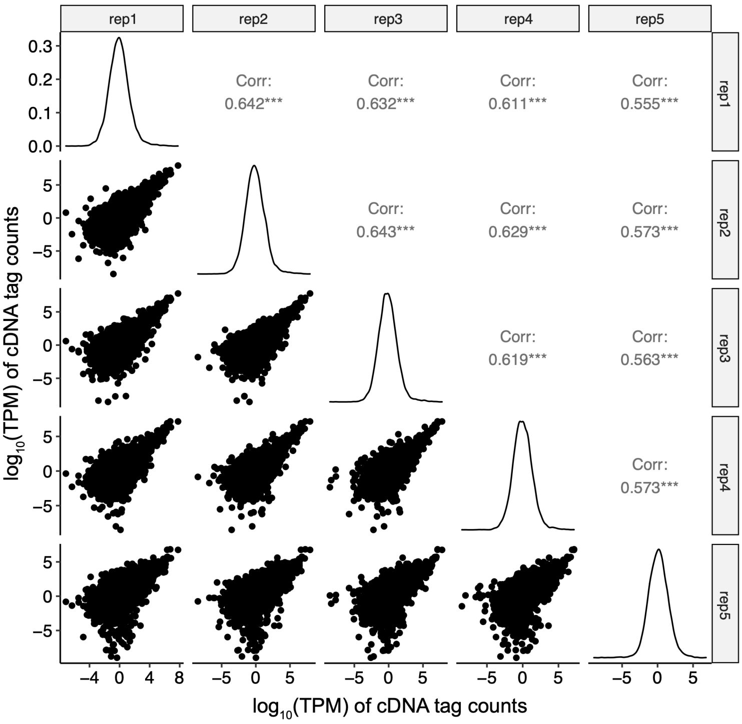

Figure S8: **Enhancer activity in caMPRA of random mutagenesis of HARs is well-correlated between replicates, Related to Fig. 2.**

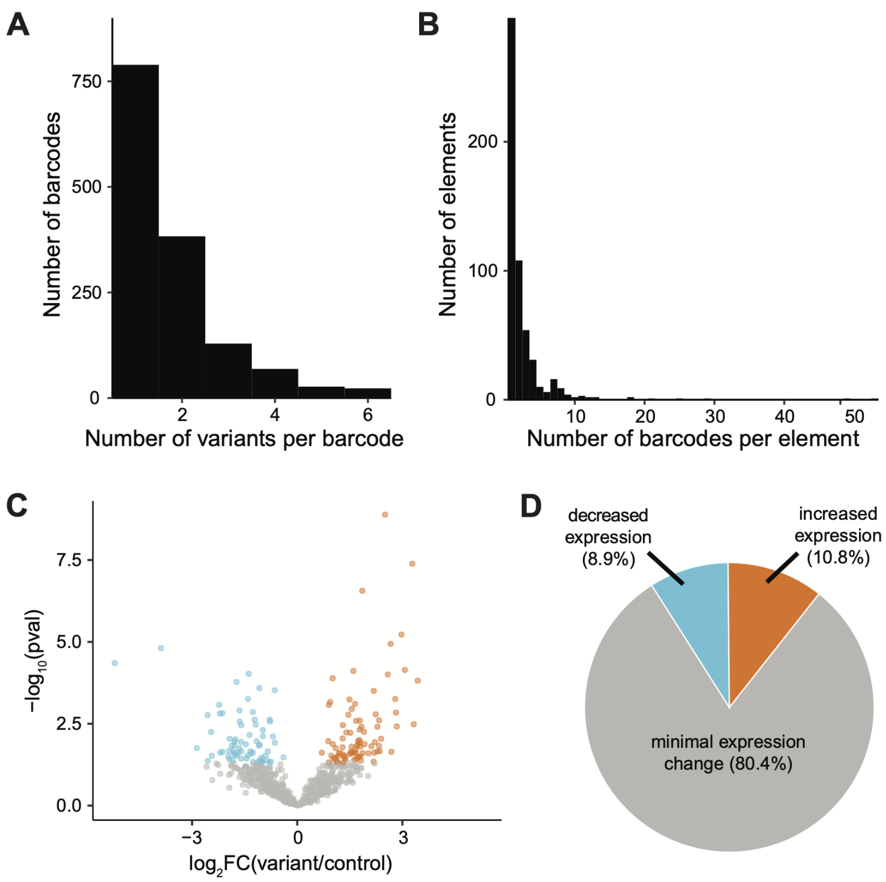

Figure S9: **Single nucleotide variants can modulate enhancer activity, Related to Fig. 2.** (A) Number of variants per mutagenized sequence (barcode). (B) Number of barcodes tested per probe. There are at least two designed probes (elements) per HAR. (C) Volcano plot of fold change in expression and adjusted p-value for barcodes that contain only one introduced variant (n = 789). (D) Pie chart of percent of barcodes with decreased expression, increased expression, or no statistically significant change in expression for barcodes that contain only one introduced variant.

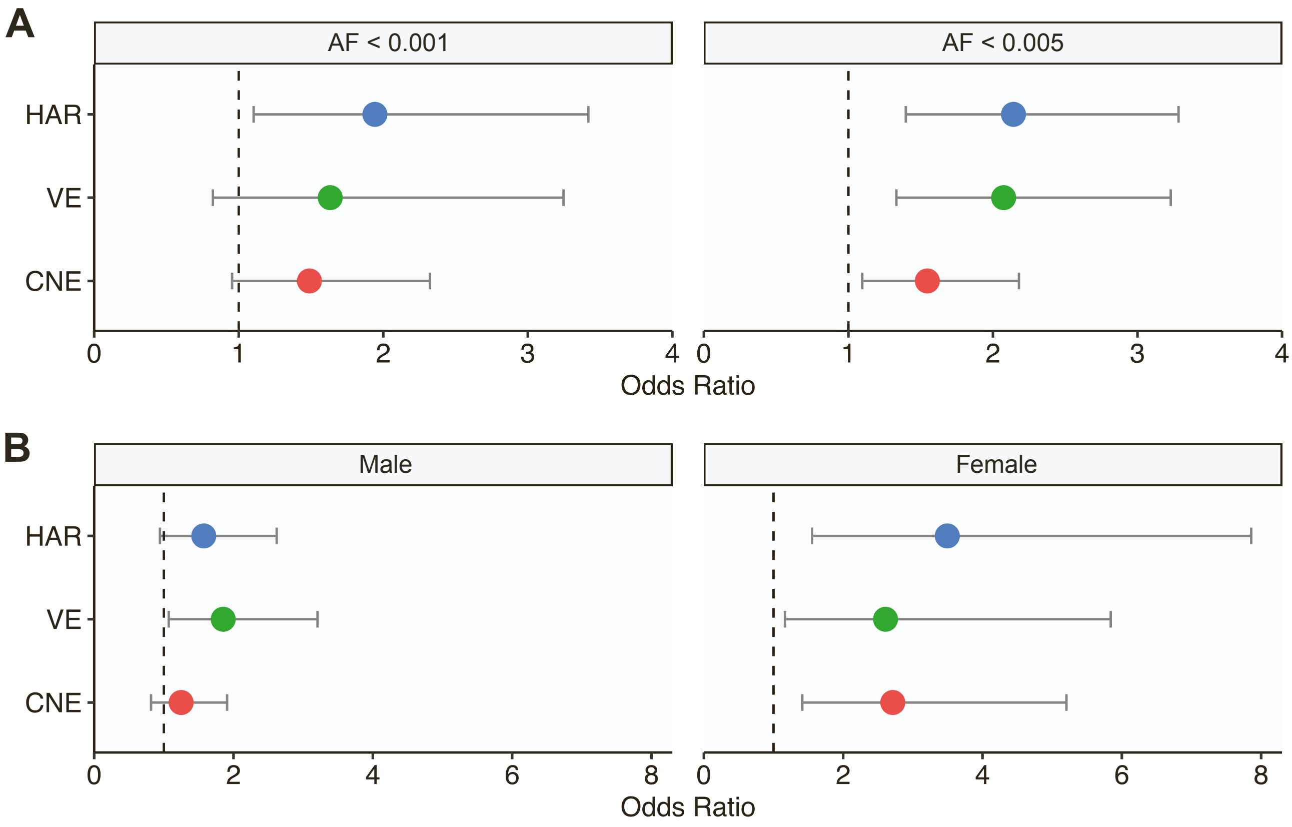

Figure S10: **Odds ratios for rare, recessive variants at conserved bases in cases versus controls in HMCA cohort are consistent across allele frequencies (A) and sexes (B), Related to Fig. 3.** (B) is assessed at allele frequency (AF) < 0.005.

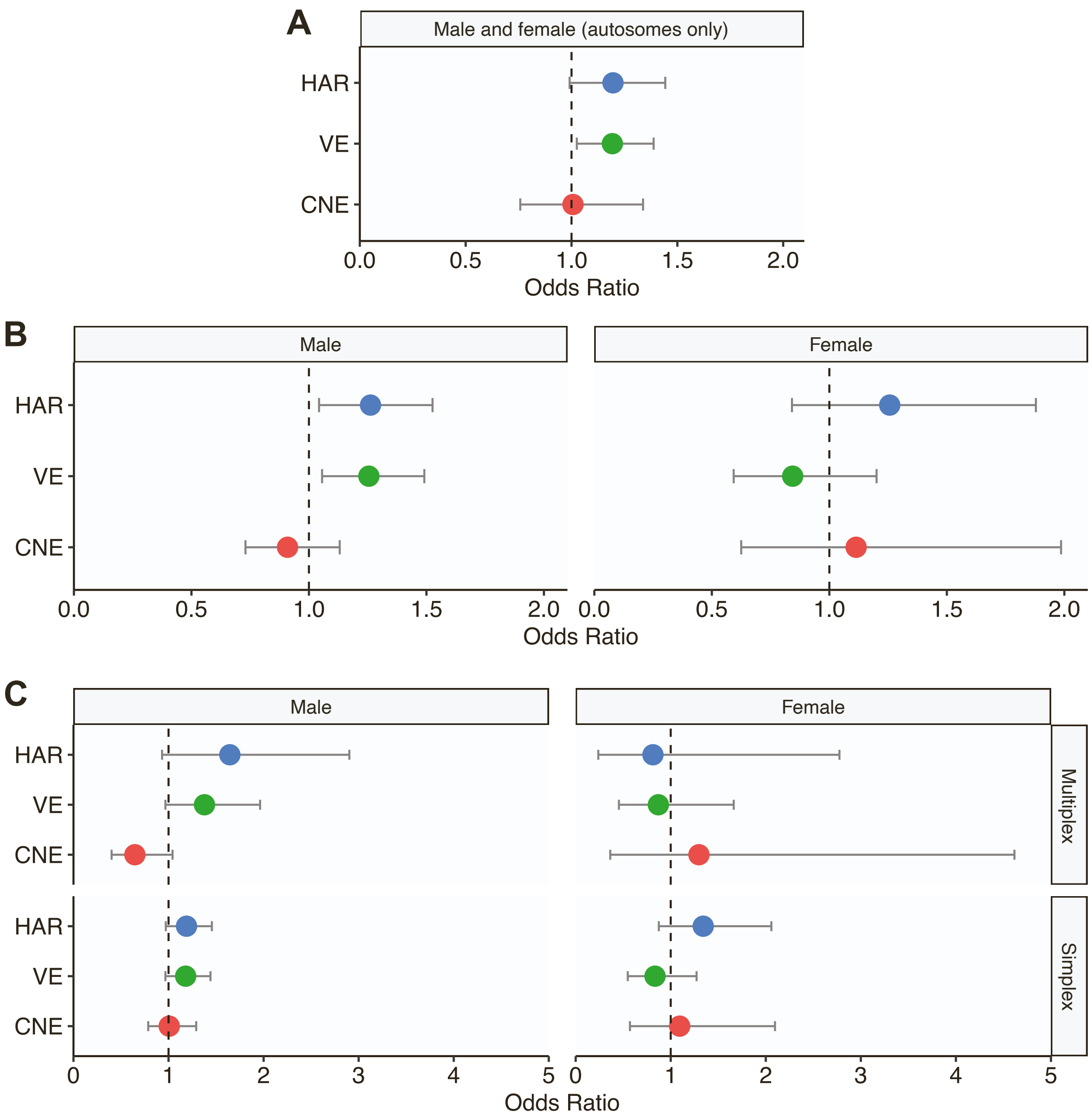

Figure S11: **Odds ratios for rare, recessive variants at conserved bases in cases versus controls in the NIMH cohort for (A) males and females (autosomes only), (B) males and females separately (autosomes and X chromosome), and (C) by family structure and sex (autosomes and X chromosome), Related to Fig. 3.** All are assessed at allele frequency (AF) < 0.001.

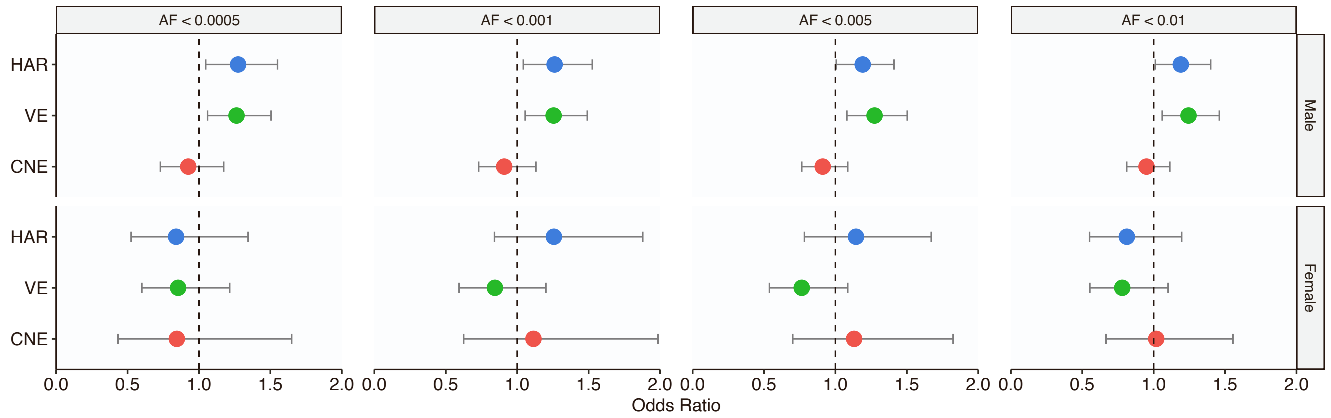

Figure S12: **Odds ratios for rare, recessive variants at conserved bases in cases versus controls in NIMH cohort are consistent across allele frequencies (AF), Related to Fig. 3.**

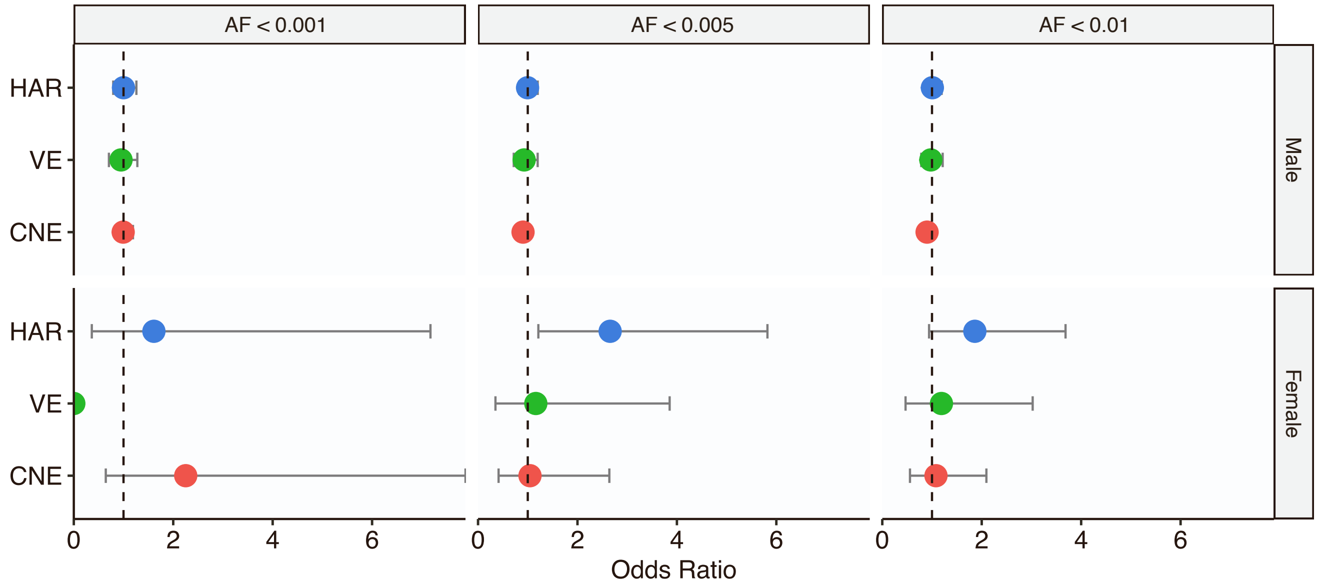

Figure S13: **Odds ratios for rare, recessive variants at conserved bases in cases versus controls in SSC cohort are consistent across allele frequencies (AF), Related to Fig. 3.**

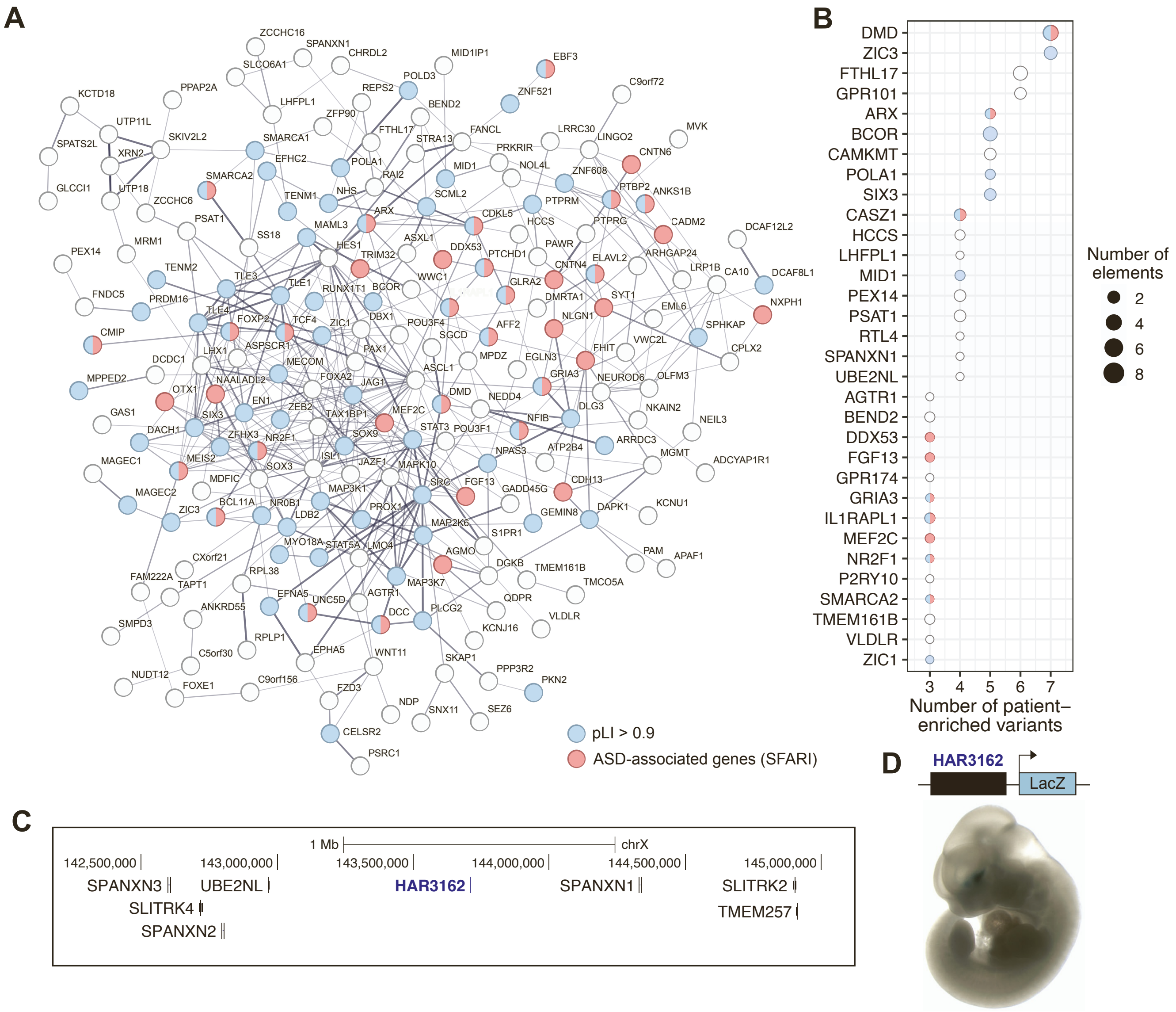

Figure S14: **Analysis of patient-enriched variants identified in the HMCA and NIMH cohorts, Related to Fig. 3.** (A) Protein-protein interactions of genes near HARs, VEs, and CNEs in the HMCA cohort and HARs and VEs in the NIMH cohort that have a numerical excess of variants found in cases compared to controls (STAR Methods). Genes associated with ASD ([S5]) are colored red, and genes that are loss-of-function intolerant (pLI > 0.9) ([S8]) are colored blue. The thickness of network edges indicates the strength of data supporting the interaction, and only networks with >5 proteins were included. (B) The number of variants found in more cases than controls (patient-enriched variants) and the number of HARs, VEs, and CNEs that they are found in (elements) are plotted for genes near at least 3 patient-enriched variants. Only patient-enriched variants where the HAR, VE, or CNE they are located in has more patient-enriched than control-enriched variants are included. Genes associated with ASD ([S5]) are colored red, and genes that are loss-of-function intolerant (pLI > 0.9) ([S8]) are colored blue. (C) Genomic interval including HAR3162 and nearby genes. (D) HAR3162 cloned upstream of a minimal promoter driving the lacZ gene was integrated at the safe-harbor H11 locus and analyzed for lacZ expression at E11.5 (STAR Methods). HAR3162 drives lacZ expression in the ventral telencephalon (representative embryo shown here).

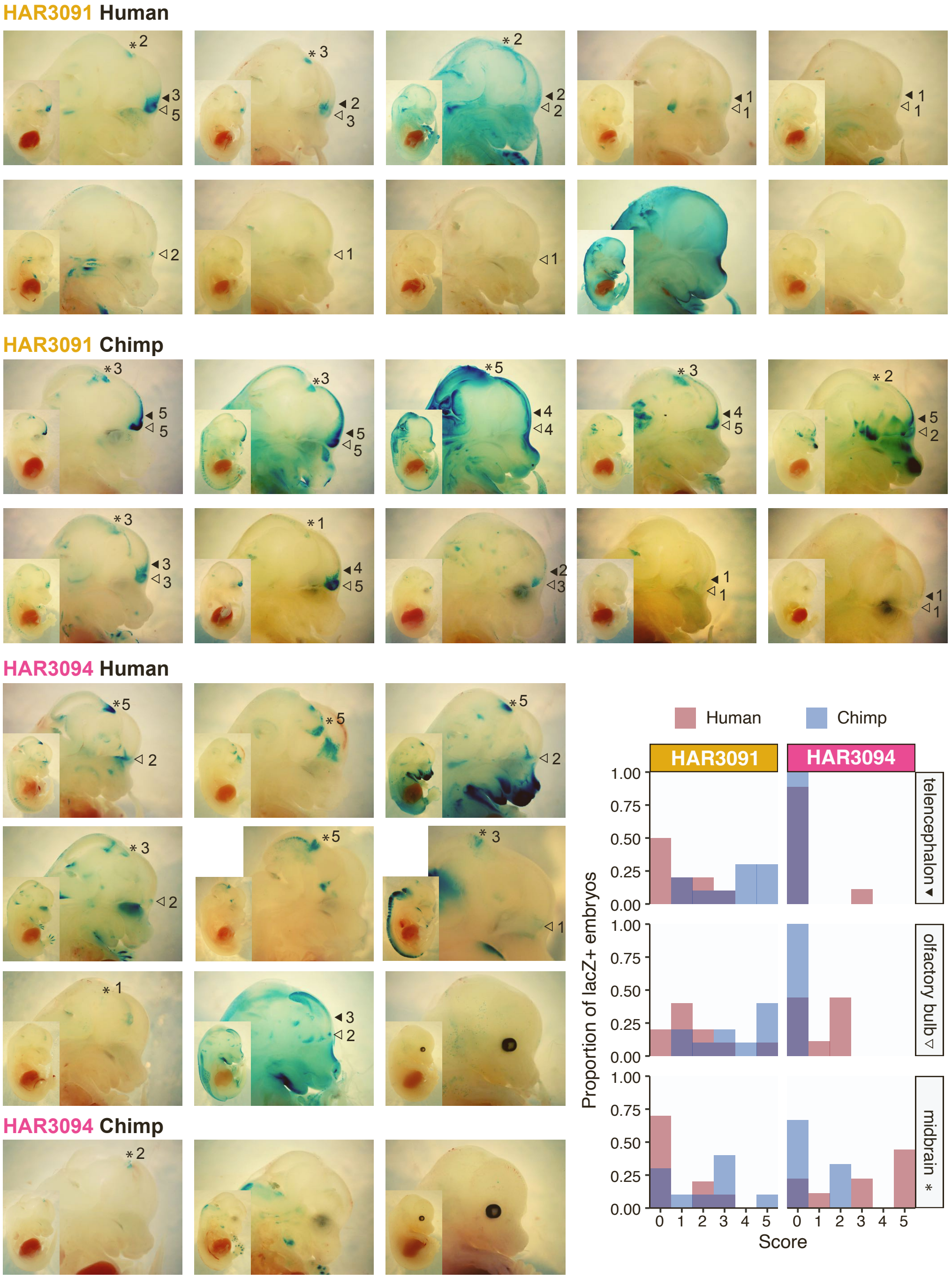

Figure S15: **Enhancer reporter assay of HAR3091 and HAR3094 in transgenic mice, Related to Fig. 4.** Enhancer reporter constructs containing the human or chimpanzee versions of HAR3091 and HAR3094 cloned upstream of a minimal promoter driving *lacZ* expression were injected into mouse embryos and analyzed at E14.5 (STAR Methods). Embryos were genotyped for the *lacZ* gene from tail clips. There were 16 PCR-positive embryos for the human version of HAR3091, 14 PCR-positive embryos for the chimpanzee version of HAR3091, 15 PCR-positive embryos for the human version of HAR3094, and 10 PCR-positive embryos for the chimpanzee version of HAR3094. All embryos with any visible lacZ staining, which is taken as a proxy that the full construct was integrated and is assessable, are displayed. Images are of bisected embryos, unless there was no internal lacZ staining. Given the mosaic and random nature of integration events in this experiment, tissue regions where the tested sequences can drive enhancer activity will show staining in multiple embryos. Each embryo was scored for enhancer activity in the telencephalon (filled arrowhead), olfactory bulb (unfilled arrowhead), and midbrain (asterisk) as follows: 5 - strong expression in many cells; 4 - strong expression in a few cells; 3 - moderate expression in many cells; 2 - moderate expression in a few cells; 1 - weak expression; 0 - no expression. E14.5 embryos have an average crown-rump length of 12 mm.

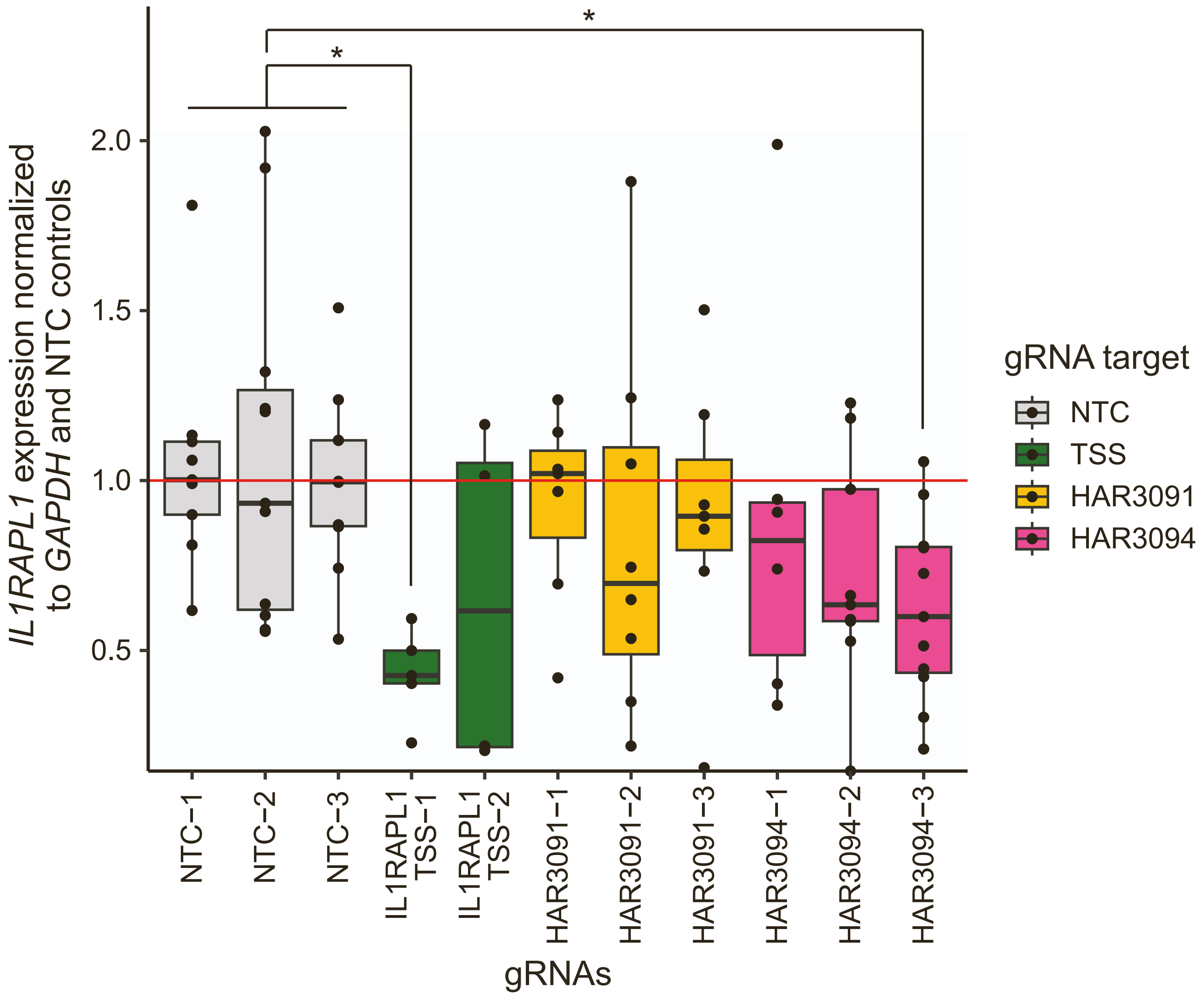

Figure S16: **CRISPRi targeting the *IL1RAPL1* promoter, HAR3091, and HAR3094, Related to Fig. 4.** We tested three non-targeting control (NTC) gRNAs (gray), two gRNAs targeting the *IL1RAPL1* promoter (green), 3 gRNAs targeting HAR3091 (yellow), and 3 gRNAs targeting HAR3094 (pink). Compared to the NTC gRNAs, only the gRNAs IL1RAPL1 TSS-1 (adjusted $p = 0.0002$) and HAR3094-7 (adjusted $p = 0.002$) were statistically significant.

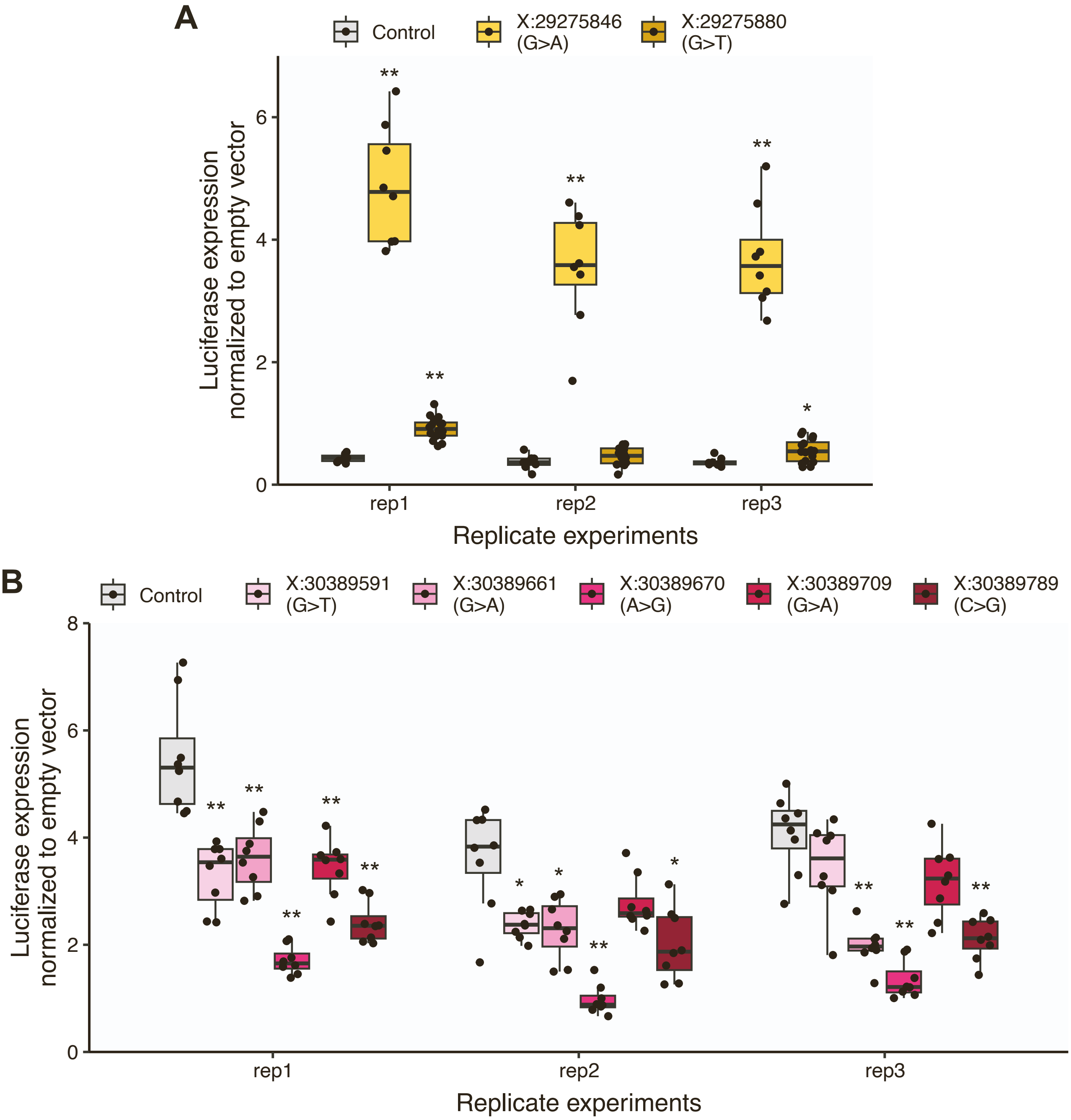

Figure S17: **Luciferase assays of patient variants in HAR3091 (A) and HAR3094 (B), Related to Fig. 4.** Within each replicate experiment, each patient sequence was compared to the luciferase expression from the control sequence with the Wilcoxon rank-sum test. P-values were adjusted using the Benjamini-Hochberg correction. * : adjusted $p < 0.1$, ** : adjusted $p < 0.01$.

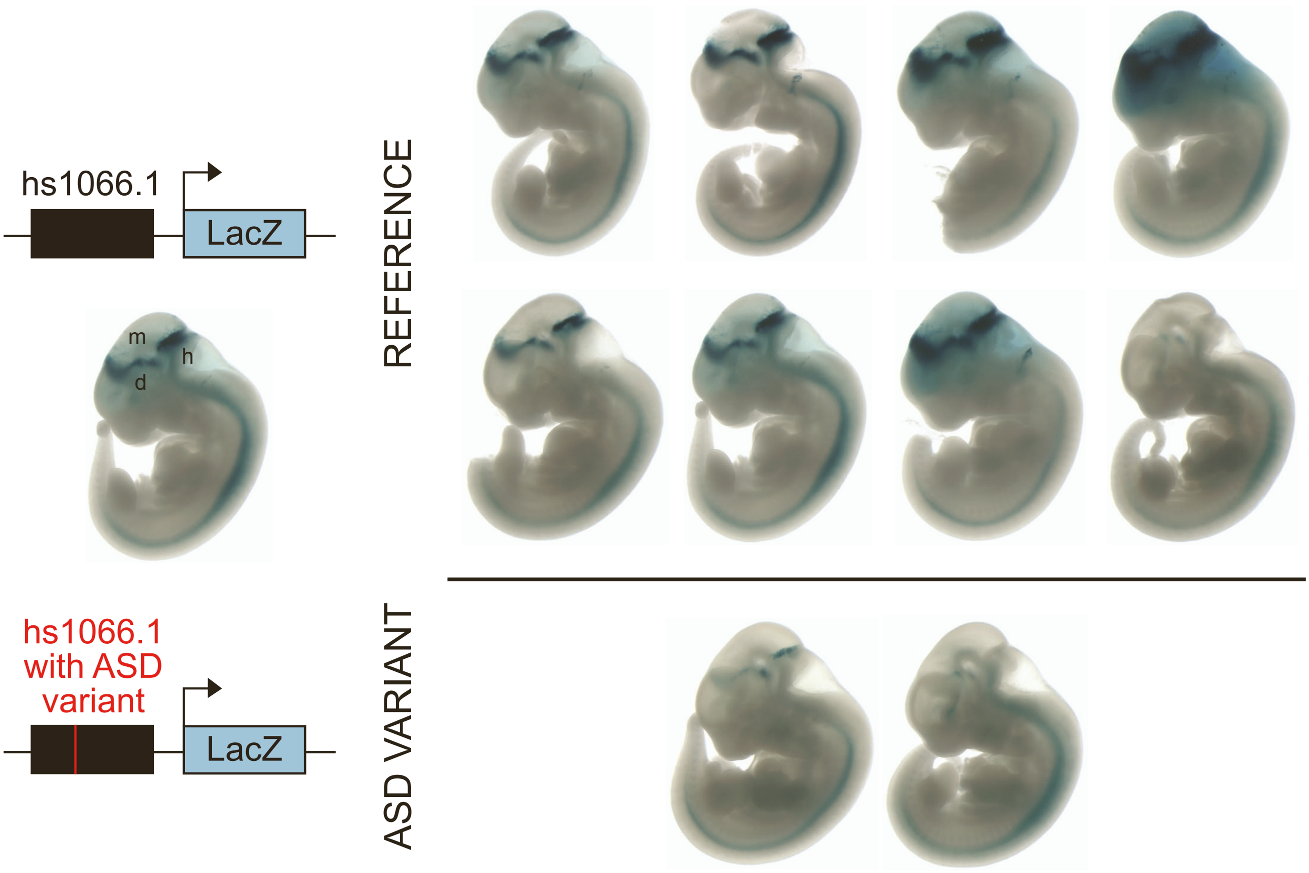

Figure S18: **Enhancer reporter assay of hs1066.1 (VE235) in E11.5 transgenic mice, Related to Fig. 5.** Enhancer reporter constructs containing hs1066.1 with or without the ASD patient variant upstream of a minimal promoter driving lacZ expression were injected into mouse embryos, screened for stable integrants at the safe-harbor H11 locus, and analyzed at E11.5 (STAR Methods). All embryos with homozygous insertions at the H11 locus are shown. E11.5 embryos have an average crown-rump length of 6 mm.

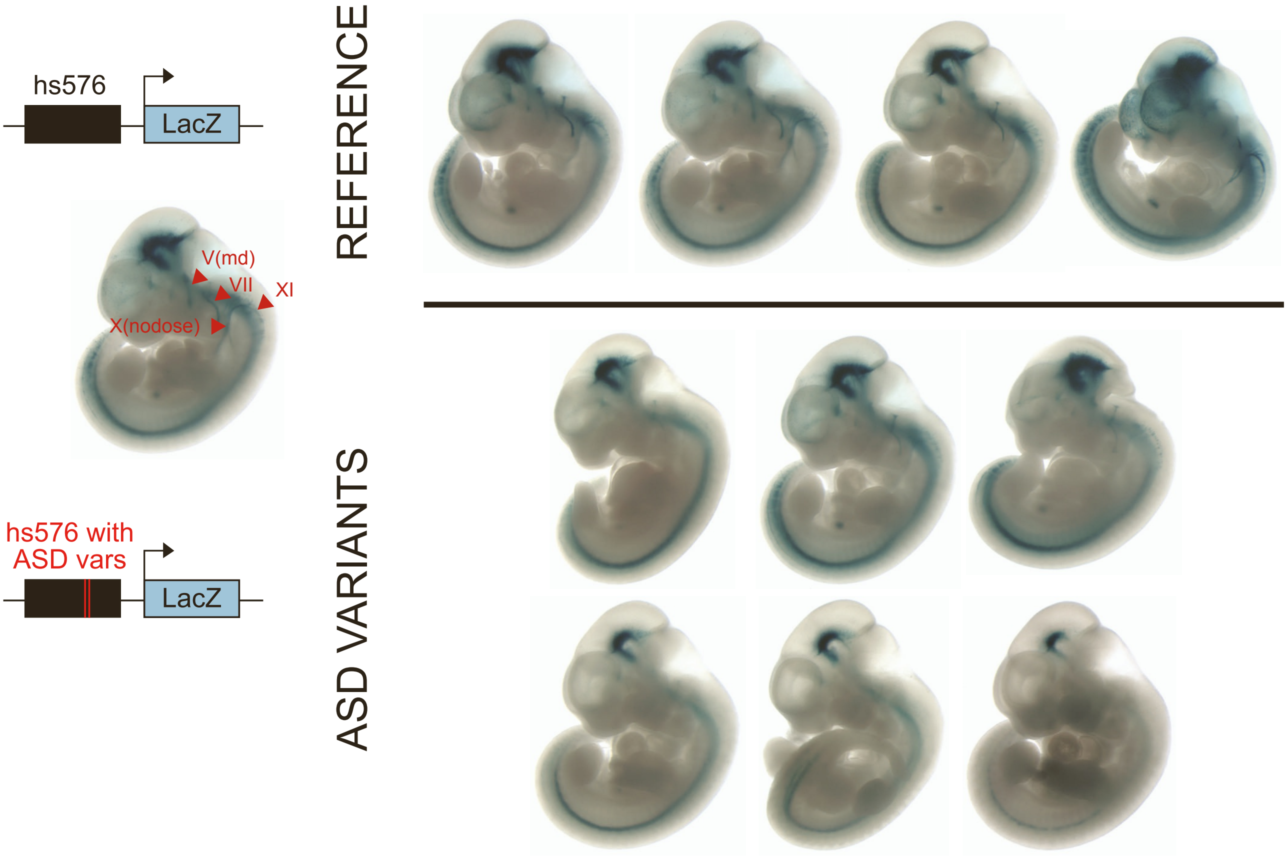

Figure S19: **Enhancer reporter assay of hs576 (VE854) in E11.5 transgenic mice, Related to Fig. 5.** Enhancer reporter constructs containing hs576 with or without the two ASD patient variants upstream of a minimal promoter driving lacZ expression were injected into mouse embryos, screened for stable integrants at the safe-harbor H11 locus, and analyzed at E11.5 (STAR Methods). All embryos with homozygous insertions at the H11 locus are shown. E11.5 embryos have an average crown-rump length of 6 mm.

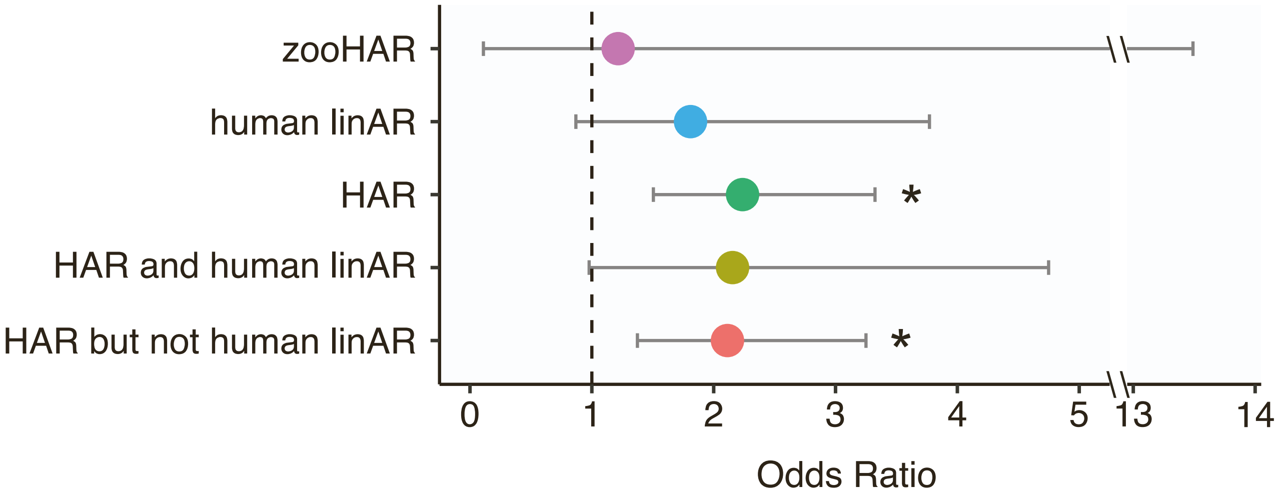

Figure S20: **Odds ratios for the number of rare, recessive variants at conserved bases in ASD cases compared to controls at allele frequency < 0.005 in the HMCA cohort for the set of HARs analyzed in this study (HAR) and two recently identified sets of HARs, zooHARs ([S9]) and human linARs ([S10]), Related to Fig. 3.** Given the moderate overlap between linARs and the HARs analyzed in this study, we also assessed HARs that overlap linARs (HAR and human linAR) and those that did not (HAR but not human linAR). Note that the genomic coverage of zooHARs and human linARs is smaller than that of the HARs assessed in this study and so we are not statistically powered to assess significance at these odds ratios.

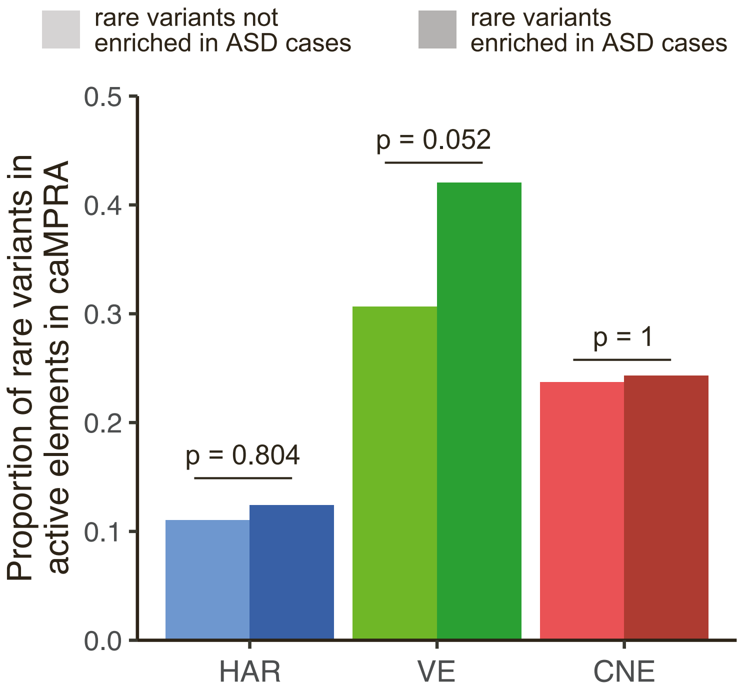

Figure S21: **Proportion of rare, recessive variants in active elements identified by caMPRA, Related to Fig. 3.** Rare, recessive variants identified in HARs, VEs, or CNEs in the HMCA cohort and in HARs or VEs in the NIMH cohort were examined. Active elements are from the D3 caMPRA experiment in Fig. 2. The denominator is the total number of elements assessed by caMPRA where at least 1 rare, recessive variant was observed. Note that there is a bias toward identifying rare variants and enhancer activity in longer elements. This bias is especially pronounced in VEs, which have the largest distribution of element lengths (Fig. S4B), and likely explains why the proportion of rare variants in active VEs is higher than the proportion of active elements among all VEs (Fig. 2B).

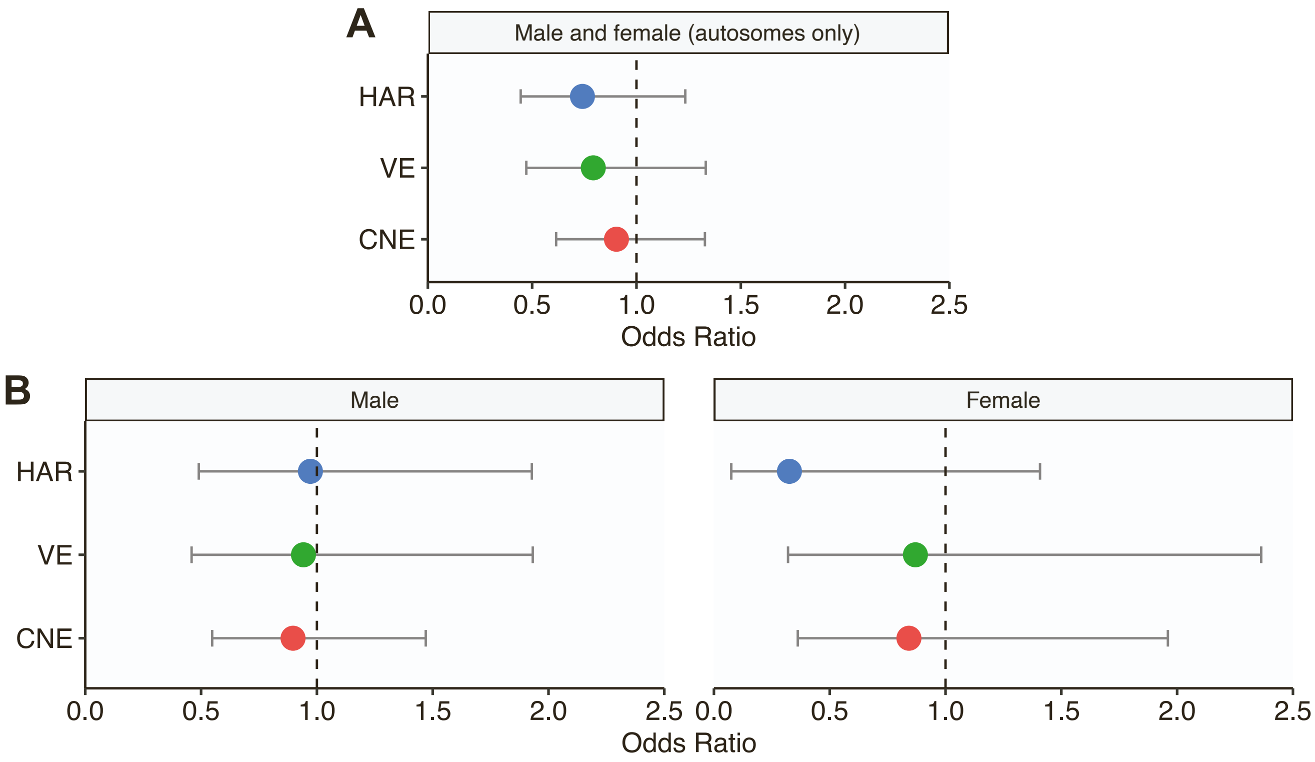

Figure S22: ***De novo*** **variants are not enriched in cases versus controls in SSC for (A) males and females (autosomes only) or (B) males and females separately (autosomes and X chromosome), Related to Fig. 3.**

Name	Sequence	Use
MlyI-F	TCCTGACACCTTCCAGCATG	Amplification of MIPs
MlyI-R	AGTCCGCAGAGATGTCCAGG	Amplification of MIPs
SfiI_half_full-F	ACTGGCCGCTTCACTG	Amplication of captures for mutagenesis
SfiI_half_full-R	CGACGCTCTTCCGATCT	Amplication of captures for mutagenesis
SfiI-F	GCTAAGGGCCTAACTGGCCGCTTCACTG	Amplification of captures
SfiI-R	GTTTAAGGCCTCCGTGGCCGACGCTCTT CCGATCT	Amplification of captures
caMPRA-Mut-F-enr	GCTAAGGGCCTAACTGGCC	Amplication of mutagenized sequences
caMPRA-Mut-R-enr	GTTTAAGGCCTCCGTGGC	Amplication of mutagenized sequences
caMPRA_SfiI-F	GCTAAGGGCCTAACTGGCCGCTTCACTG	Mutagenesis of captures
Mutagenesis-full-R	GTTTAAGGCCTCCGTGGCCGACGCTCTT CCGATCTnnnnnNNNNNnnnnnNNNNN nnnnnGCGATCGCCCTCGAGG	Mutagenesis of captures
IL1RAPL1-qRTPCR-F	GCTGAAGAGCTCGATGGAGA	qPCR
IL1RAPL1-qRTPCR-R	TCCACTGGTCAGGATCCACT	qPCR
GAPDH-qRTPCR-F	GAACGGGAAGCTTGTCATCAA	qPCR
GAPDH-qRTPCR-R	ATCGCCCCACTTGATTTTGG	qPCR
NTC2 gRNA	TGTGCAACCTCCGCCGTTG	CRISPRi
NTC3 gRNA	CCCGAGCAGTGGCTCGCTA	CRISPRi
NTC5 gRNA	GAGGACGATCGTACTCCAG	CRISPRi
IL1RAPL1 TSS-1 gRNA	AACCGATCTTGTAGAAACAC	CRISPRi
IL1RAPL1 TSS-2 gRNA	CCGCAATAACAGATCCGAC	CRISPRi
HAR3091-1 gRNA	TATAAGGAGAATTAGTCCCG	CRISPRi
HAR3091-2 gRNA	ACTCTCAAAATAAATTCCCC	CRISPRi
HAR3091-3 gRNA	ACATGTTGTTATAACTTCTC	CRISPRi
HAR3094-1 gRNA	ATTATTACCCACCTACTATC	CRISPRi
HAR3094-2 gRNA	TGAAACACTGGCCTGATAGT	CRISPRi
HAR3094-3 gRNA	GTCAGTCCTTTCTGAAACAC	CRISPRi

Table S5: **Oligos used in this study, Related to STAR Methods.**

References

[S1] Osterwalder, M., Barozzi, I., Tissieres, V., Fukuda-Yuzawa, Y., Mannion, B.J., Afzal, S.Y., Lee, E.A., Zhu, Y., Plajzer-Frick, I., Pickle, C.S., et al. (2018). Enhancer redundancy provides phenotypic robustness in mammalian development. Nature *554*, 239–243.

[S2] Visel, A., Minovitsky, S., Dubchak, I., and Pennacchio, L.A. (2007). VISTA Enhancer Browser–a database of tissue-specific human enhancers. Nucleic Acids Res *35*, D88-92.

[S3] Kundaje, A., Meuleman, W., Ernst, J., Bilenky, M., Yen, A., Heravi-Moussavi, A., Kheradpour, P., Zhang, Z., Wang, J., Ziller, M.J., et al. (2015). Integrative analysis of 111 reference human epigenomes. Nature *518*, 317–330.

[S4] McLean, C.Y., Bristor, D., Hiller, M., Clarke, S.L., Schaar, B.T., Lowe, C.B., Wenger, A.M., and Bejerano, G. (2010). GREAT improves functional interpretation of cis-regulatory regions. Nat Biotechnol *28*, 495–501.

[S5] Abrahams, B.S., Arking, D.E., Campbell, D.B., Mefford, H.C., Morrow, E.M., Weiss, L.A., Menashe, I., Wadkins, T., Banerjee-Basu, S., and Packer, A. (2013). SFARI Gene 2.0: a community-driven knowledgebase for the autism spectrum disorders (ASDs). Mol Autism *4*, 36.

[S6] Karczewski, K.J., Francioli, L.C., Tiao, G., Cummings, B.B., Alföldi, J., Wang, Q., Collins, R.L., Laricchia, K.M., Ganna, A., Birnbaum, D.P., et al. (2020). The mutational constraint spectrum quantified from variation in 141,456 humans. Nature *581*, 434–443.

[S7] Zhou, J., and Troyanskaya, O.G. (2015). Predicting effects of noncoding variants with deep learning–based sequence model. Nat Methods *12*, 931–934.

[S8] Lek, M., Karczewski, K.J., Minikel, E.V., Samocha, K.E., Banks, E., Fennell, T., O'Donnell-Luria, A.H., Ware, J.S., Hill, A.J., Cummings, B.B., et al. (2016). Analysis of protein-coding genetic variation in 60,706 humans. Nature *536*, 285–291.

[S9] Keough, K.C., Whalen, S., Inoue, F., Przytycki, P.F., Fair, T., Deng, C., Steyert, M., Ryu, H., Lindblad-Toh, K., Karlsson, E., et al. (2023). Three-dimensional genome rewiring in loci with human accelerated regions. Science *380*, eabm1696.

[S10] Bi, X., Zhou, L., Zhang, J.-J., Feng, S., Hu, M., Cooper, D.N., Lin, J., Li, J., Wu, D.-D., and Zhang, G. (2023). Lineage-specific accelerated sequences underlying primate evolution. Science Advances *9*, eadc9507.
